# Integrative Metabolomic and Lipidomic Profiling of Lung Squamous Cell Carcinoma for Characterization of Metabolites and Intact Lipid Species Related to the Metastatic Potential

**DOI:** 10.3390/cancers13164179

**Published:** 2021-08-19

**Authors:** Heayyean Lee, Hwanhui Lee, Sujeong Park, Myeongsun Kim, Ji Young Park, Hanyong Jin, Kyungsoo Oh, Jeehyeon Bae, Young Yang, Hyung-Kyoon Choi

**Affiliations:** 1 College of Pharmacy, Chung-Ang University, Seoul 06974, Korea; hyeonlee38@gmail.com (H.L.); hwanhui56@gmail.com (H.L.); myeongsunkim0242@gmail.com (M.K.); kyungsoooh@cau.ac.kr (K.O.); jeehyeon@cau.ac.kr (J.B.); 2 Department of Biological Sciences, Sookmyung Women’s University, Seoul 04312, Korea; psj8718@sookmyung.ac.kr (S.P.); pakgy1018@gmail.com (J.Y.P.); 3 Department of Life Science, Chung-Ang University, Seoul 06974, Korea; hanyong1985@naver.com

**Keywords:** lung squamous cell carcinoma, metastatic potential, metabolomics, lipidomics, GC-MS, DI-MS

## Abstract

**Simple Summary:**

Non-small cell lung cancer (NSCLC) is the most severe cancer showing a low 5-year survival rate of metastatic NSCLC, but there are few previous reports for prediction of metastatic potential and identification of therapeutic targets of lung squamous cell carcinoma (SQCC), a major type of NSCLC, with different metastatic potential based on metabolic and lipidomic profiling. We identified metabolites and intact lipid species relevant to lung SQCC metastatic potential, which could be applied to develop potential biomarkers and therapeutic targets.

**Abstract:**

SQCC is a major type of NSCLC, which is a major cause of cancer-related deaths, and there were no reports regarding the prediction of metastatic potential of lung SQCC by metabolomic and lipidomic profiling. In this study, metabolomic and lipidomic profiling of lung SQCC were performed to predict its metastatic potential and to suggest potential therapeutic targets for the inhibition of lung SQCC metastasis. Human bronchial epithelial cells and four lung SQCC cell lines with different metastatic potentials were analyzed using gas chromatography–mass spectrometry and direct infusion-mass spectrometry. Based on the obtained metabolic and lipidomic profiles, we constructed models to predict the metastatic potential of lung SQCC; glycerol, putrescine, β-alanine, hypoxanthine, inosine, *myo*-inositol, phosphatidylinositol (PI) 18:1/18:1, and PI 18:1/20:4 were suggested as characteristic metabolites and intact lipid species associated with lung SQCC metastatic potential. In this study, we established predictive models for the metastatic potential of lung SQCC; furthermore, we identified metabolites and intact lipid species relevant to lung SQCC metastatic potential that may serve as potential therapeutic targets for the inhibition of lung SQCC metastasis.

## 1. Introduction

Non-small cell lung cancer (NSCLC) is the leading cause of cancer-related deaths worldwide [1]. In particular, metastasis is a major cause of death attributed to various types of cancer, including NSCLC, and accounts for about 90% of all cancer cases [2,3]. Early stage non-metastatic NSCLC can be successfully treated through surgical resection and chemotherapy. However, a significant proportion of NSCLC patients undergo cancer relapse with metastasis after surgical resection [4,5]. Moreover, nearly 55% of all NSCLC cases are only diagnosed after the detection of a locally advanced or metastatic tumor [6]. When NSCLC spread to distinct parts of the body, called metastatic NSCLC, the 5-year survival rate is less than 10% [7]. Therefore, an identifiable biomarker for metastasis prediction and improved knowledge of the biological mechanisms controlling metastatic potential are urgently needed for effective treatment and increasing survival rates of NSCLC.

Among the various types of NSCLC, lung squamous cell carcinoma (SQCC) is the second most common, following adenocarcinoma (AC) [8]. Lung SQCC has histological characteristics different from those of other types of NSCLC; it arises most frequently in larger airways, whereas lung AC and large cell carcinomas occur most frequently in the lung periphery [9]. Moreover, lung SQCC shows a genomic pattern distinct from that of lung AC; epidermal growth factor receptor and Kirsten rat sarcoma viral racial oncogene mutations are typically present in lung AC but not in lung SQCC [10]. In contrast, TP53 somatic mutations are primarily observed in lung SQCC patients but not in lung AC patients [11]. Due to differences in their histological and genomic characteristics, lung SQCC is likely to be associated with different biological metabolic pathways and prognostic factors, compared with other types of NSCLC. Therefore, understanding the biological mechanisms of lung SQCC metastasis may uncover a suitable method for effectively inhibiting and treating metastasis.

Cancer metabolism has recently gained major attention in cancer research, and metabolomics and lipidomics studies have provided comprehensive information to improve our understanding of cancer pathogenesis [12,13,14]. As cancer progresses, metabolomic analysis can be used to accurately analyze the changes in the phenotype and associated biological mechanisms of the tumor, because metabolites are the final downstream products of genes that are differentially expressed in cancer cells [15]. However, there have been no reports on the prediction of lung SQCC metastasis through metabolomic and lipidomic analyses. An integrated study of comprehensive metabolite profiling and intact lipid species profiling for lung SQCC metastasis could provide a systems-level perspective toward the development of novel therapeutic targets.

In this study, we performed comprehensive metabolite profiling and intact lipid species profiling of primary human bronchial epithelial cells (HBEpC), and lung SQCC cell lines (H520, HCC95, SK-MES-1, and H1703), using gas chromatography–mass spectrometry (GC-MS) and direct infusion-mass spectrometry (DI-MS). We hypothesized that analyzing the metabolites and intact lipid species in lung SQCC cells with varying metastatic potentials could provide information regarding lung SQCC-specific biomarkers to predict metastasis and identify biological mechanisms that are activated during cancer progression. Therefore, the aim of this study was to discover lung SQCC-specific metabolic biomarkers that may be useful for inclusion in a lung SQCC metastatic potential prediction model and to reveal the biological mechanisms of lung SQCC metastasis based on integrated metabolomic and lipidomic analyses.

## 2. Materials and Methods

### 2.1. Chemicals and Reagents

High-performance liquid chromatography (HPLC)-grade methanol was obtained from Thermo Fisher Scientific (Hampton, NH, USA). HPLC-grade hexanes were obtained from Honeywell Burdick & Jackson (Muskegon, MI, USA). Myristic-d_27_ acid, methoxylamine hydrochloride, and pyridine were obtained from Sigma Aldrich (St. Louis, MO, USA). N,O-bis(trimethylsilyl) trifluoroacetamide containing 1% trimethylchlorosilane was obtained from Alfa Aesar (Ward Hill, MA, USA). 

### 2.2. Cell Culture and Sample Collection

The human lung SQCC cell lines, H520 was obtained from the American Type Culture Collection (ATCC; Manassas, VA, USA), and HCC95, H1703, and SK-MES-1 were obtained from the Korean Cell Line Bank (Seoul, Korea), and cultured in RPMI 1640 medium (H520, HCC95, H1703) and Dulbecco’s modified Eagle’s medium (SK-MES-1) supplemented with 10% heat-inactivated fetal bovine serum and 1% penicillin–streptomycin (Hyclone, Logan, UT, USA). The HBEpC cells were obtained from PromoCell GmbH (Heidelberg, Germany) and cultured in an airway epithelial cell growth medium containing 2.46% SupplementMix (PromoCell GmbH). Cell culture was performed as previously described [16]. Each cell line was cultured in four biological replicates.

### 2.3. Cell Migration, Invasion, and Proliferation Assay

Migration and invasion assays were performed three times independently using Transwell permeable supports (8 µm pore size, 6.5 mm insert; Corning Inc., Corning, NY, USA) as previously described [16]. Cells were plated at a density of 1 × 10^5^ cells/well. After 24 h incubation, cells were fixed and stained with crystal violet (Sigma Aldrich). The stained cells were solubilized with 20% methanol and the optical density at 590 nm (OD590) was measured using the FlexStation 3 Microplate Reader (Molecular Devices, San Jose, CA, USA).

Cell proliferation was measured by using a BrdU cell proliferation colorimetric ELISA Kit (ab126556, Abcam, Cambridge, MA, USA) following the manufacturer’s protocol. Briefly, cells were seeded into 96-well plates, incubated with 20 µL of 1× BrdU reagent at 37 °C and 5% CO_2_ for 16 h and fixed by provided solution. Fixed cells were incubated with anti-BrdU monoclonal detector antibody, 1× peroxidase goat anti-mouse IgG conjugate, and TMB substrate. BrdU incorporation was measured at 450 nm and 540 nm.

### 2.4. Immunoblotting Analysis 

For immunoblotting, cells were prepared in lysis buffer (50 mM Tris-HCl pH 8.0, 150 mM NaCl, 1 mM EDTA, 1% NP-40, and a protease inhibitors cocktail) and centrifuged for 15 min at 4 °C at 13,500 rpm. Whole lysates were mixed with 5× SDS buffer. Proteins were subjected to SDS-PAGE gel electrophoresis and transferred to 0.45 µm nitrocellulose membrane. Blocked membranes with 3% BSA were incubated with primary antibody at 4 °C for overnight. Following washing with TBS-T, HRP-conjugated secondary antibody was incubated for 2 h at room temperature. The proteins were visualized with ECL solution and detected using a luminescent image analyzer (Fuji Film, Tokyo, Japan). Primary antibodies used for immunoblotting were as follows: anti-β-actin (Santa Cruz, CA, USA; sc-477778), anti-vimentin (Cell Signaling, MA, USA; 3932S), anti-E-cadherin (Santa Cruz, sc-8426), anti-AKT (Cell Signaling, 4691S), and anti-*p*-AKT(T308) (Cell Signaling, 9275S).

### 2.5. RT-PCR Analysis

For RNA preparation, 500 µL TRIzol was added to cell culture plates. Cells were detached by using a cell scraper and mixed with 100 µL chloroform. Following centrifugation, 200 µL of the supernatant was transferred to a new tube, mixed with an equal volume of isopropanol, and centrifuged at 13,500 rpm for 15 min at 4 °C. The RNA pellet was resuspended in 20 µL of ultra-pure distilled water (Invitrogen, CA, USA). Purified RNA was reverse transcribed using the RevertAid RT Reverse Transcription Kit (Thermo Fisher Scientific, Inc., Fermetas, MA, USA). Reverse transcription–quantitative polymerase chain reaction (RT-qPCR) was carried out using ExcelTaq 2X Q-PCR Master Mix (SMOBIO, Hsinchu, Taiwan). RT-PCR data were normalized with the primers used for RT-qPCR amplification, and primer sequences were listed in Table S1.

### 2.6. GC-MS Analysis

To determine the protein concentration, protein assays were performed as previously described [16]. For metabolite extraction, 700 µL ice-cold methanol (100%, HPLC grade) was added to freeze-dried cells and vortexed for 30 s. The mixture was sonicated at 4 °C for 30 min and then centrifuged at 3000× *g* for 3 min at 4 °C. The supernatant was transferred into Eppendorf tubes and filtered through 0.45 μm polytetrafluoroethylene syringe filters (Membrane Solutions, Kent, WA, USA). Next, 400 μL filtrate was transferred into GC vials, derivatized, and profiled using GC-MS, as previously described [16]. The experiment was performed twice with four biological replicates.

For analysis, the initial oven temperature was set at 70 °C and increased to 190 °C (5 °C/min), 240 °C (6 °C/min), 270 °C (5 °C/min), and finally to 280 °C (3 °C/min). Electron multiplier voltage was set at 1141 V. Cellular metabolites were assigned through comparison with data in the Nist-Wiley Mass Spectra Library, the Human Metabolome Database (HMDB; http://www.hmdb.ca/ accessed on 15 Jan 2021), and the Golm Metabolome Database (GMD; gmd.mpimp-golm.mpg.de/ accessed on 15 Jan 2021).

### 2.7. DI-MS Analysis

For lipid profiling, lipids were extracted and analyzed using DI-MS, as previously described [16]. Each sample was prepared in four biological replicates and the experiment was performed twice. The mass spectrometer was set at a capillary voltage of 35 V in positive ion mode and −45 V in negative ion mode, tube lens voltage was set at 130 V in positive ion mode, and −118 V in negative ion mode, and the capillary temperature was set at 200 °C. For the identification of lipid species, LipidBlast by Kind [17], Lipidmaps (http://www.lipidmaps.org/ accessed on 28 January 2021), and in-house MS/MS library databases were used. Additionally, MS/MS spectra of authentic reference were used for the identification of ceramide species [18].

### 2.8. Data Processing and Statistical Analysis

The raw data files were processed, as previously described [16]. The resulting datasets were used to construct a heatmap with hierarchical clustering and pathway analysis with MetaboAnalyst (version 5.0; https://www.metaboanalyst.ca accessed on 20 May 2021) [19]. For heatmap clustering, the resulting datasets were converted to *z*-score. The row *z*-score was calculated as the mean value subtracted from the relative level of each compound and then divided by the standard deviation of the relative level of each compound. The impact value threshold of pathway analysis was set to 0.10. Significant differences in the levels of each metabolite and intact lipid species were evaluated by one-way analysis of variance (ANOVA) with Tukey’s post hoc test using SPSS software (version 25; IBM, Armonk, NY, USA). For multivariate statistical analysis, the dataset composed of metabolites and lipids was mean-centered and scaled to unit variance, and principal component analysis (PCA) and partial-least-squares-discriminant analysis (PLS-DA) were performed using SIMCA software (version 15.0.2; Sartorius Stedim Data Analytics AB, Umeå, Sweden).

## 3. Results

### 3.1. Cell Migration, Invasion, Proliferation Assay, and p-AKT Expression in Lung SQCC

**Figure 1:**
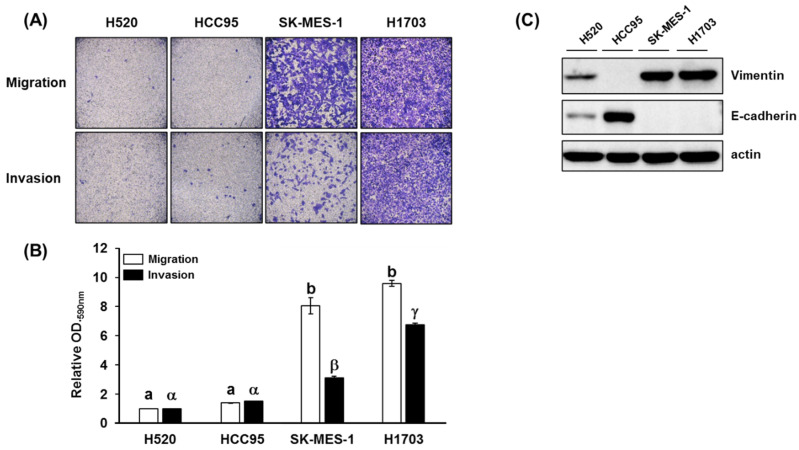
Migration and invasion properties of different lung squamous cell carcinoma (SQCC) cell lines: (**A**) representative images of cell migration and invasion in the four investigated lung SQCC cell lines; (**B**) quantified data (mean ± SEM) from three independent experiments demonstrating the migration and invasion potential of the four lung SQCC cell lines tested; (**C**) expression of vimentin and E-cadherin of four lung SQCC cell lines. The actin was used as a loading control. Images of the migrated- or invaded-cells were taken at ×100 magnification under a bright-field microscope. Different letters, (a, b) and (α, β, and γ), indicate statistically significant differences of migration and invasion levels among lung SQCC cells (*p* < 0.01). Uncropped blot image for each antibody is presented in Figure S4.

The metastatic potential, cell motility, and invasiveness of lung SQCC cell lines (H520, HCC95, SK-MES-1, and H1703) were measured by migration and invasion assay. After 24 h incubation, SK-MES-1 and H1703 displayed higher levels of migration and invasion than H520 and HCC95 (Figure 1A, B). In addition, the metastatic potential of each lung SQCC cell line was confirmed by the expression of vimentin and E-cadherin in protein and mRNA levels (Figure 1C and Figure S1). Overexpression of vimentin and loss of E-cadherin expression was correlated with increased cancer invasiveness and metastasis [20,21]. Based on these results, H520 and HCC95 were classified as lung SQCC with low-metastatic potential, whereas SK-MES-1 and H1703 were classified as lung SQCC with high-metastatic potential (Figure 1).

As shown in Figure S2, the highest cell proliferation was observed in the high-metastatic lung SQCC cell line (H1703), followed by low-metastatic lung SQCC cell lines (H520 and HCC95) and normal cell line (HBEpC). The lowest cell proliferation was observed in the SK-MES-1 cell line.

Overexpression of phosphorylated AKT (*p*-AKT) was observed in high-metastatic lung SQCC cell line (H1703), and relatively reduced expression was observed in low-metastatic lung SQCC cell lines (H520 and HCC95), as shown in Figure S3.

### 3.2. Comprehensive Metabolite and Intact Lipid Species Analyses

The metabolites and intact lipid species in HBEpC and four lung SQCC cell lines (H520, HCC95, SK-MES-1, and H1703) were comprehensively profiled. A total of 54 metabolites and 62 lipids were identified by GC-MS and DI-MS, respectively (Tables S2–S4). 

**Figure 2:**
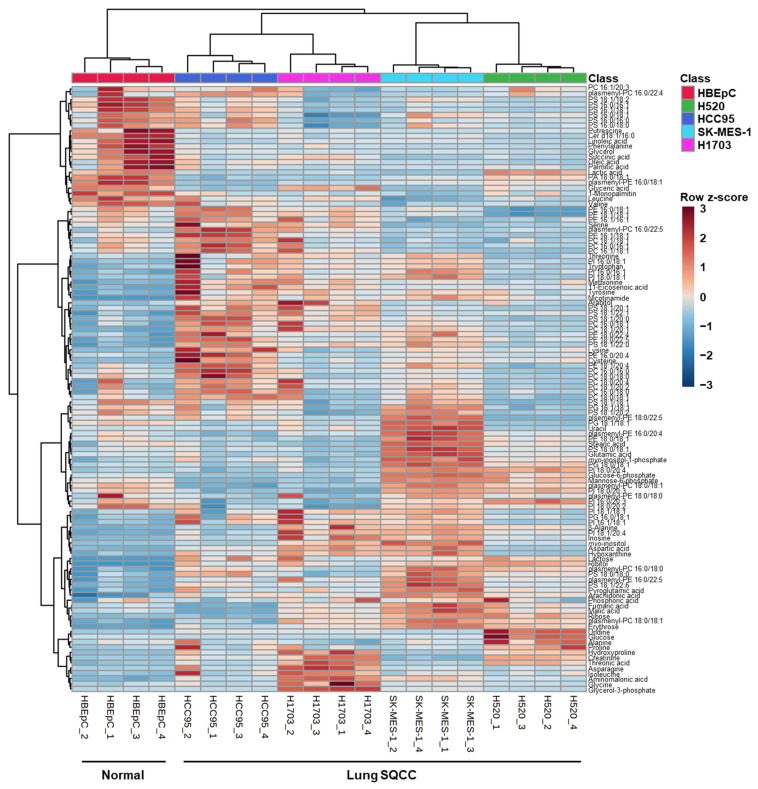
Heatmap demonstrating the relative levels of metabolites and intact lipid species in human bronchial epithelial cells (HBEpC) and four lung squamous cell carcinoma cell lines (H520, HCC95, SK-MES-1, and H1703). (*n* = 4, four biological replicates). To indicate the relative levels, the values of biological replicates were represented as mean values of technical replicates in each biological replicate. The row *z*-score for each compound was used to color code in the heatmap: low concentration compounds were colored as blue, and high concentration compounds were colored as red.

A total of 116 identified metabolites and intact lipid species were visualized by the constructed heatmap (Figure 2). The results of hierarchical clustering analysis showed that the normal group (HBEpC) and the four lung SQCC groups were clustered separately. This separation implies that the profiles of metabolites and intact lipid species in each cell line were different. An overview of the metabolic and lipidomic profiles of each cell line is represented by the metabolic and lipidomic patterns in the heatmap.

### 3.3. PLS-DA for the Prediction of Metastatic Potential of Lung SQCC

PCA was performed to visualize group separations using a total of 116 identified metabolites and lipids obtained from the metabolic and lipidomic profiling of HBEpC and four lung SQCC samples. The PCA-derived score plots showed that the normal cells and the four lung SQCC samples were clearly separated into PC 1 and PC 2, which accounted for 31.7% and 24.6% of the total variance, respectively (Figure S5).

PLS-DA was performed to maximize the intergroup separation of each sample. The variable influence on projection (VIP) values derived from the PLS-DA models reflected the influence of each component towards building the predictive model. Metabolites and intact lipid species with VIP values > 1.0 were regarded as compounds that strongly contributed to the ability to predict metastatic potential [22].

**Figure 3:**
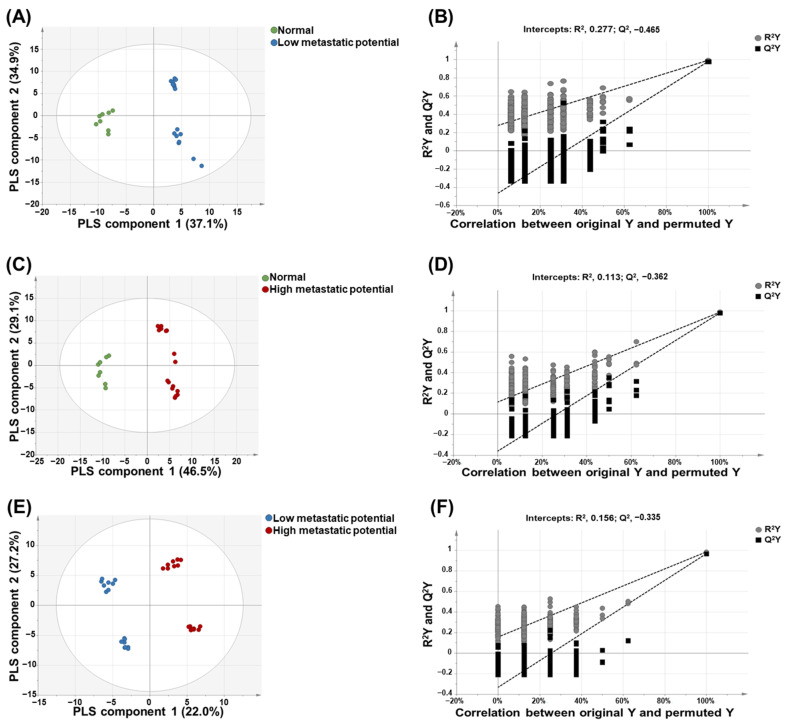
Partial least-squares discriminant analysis (PLS-DA)-derived score plots and permutation test. PLS-DA-derived score plot (**A**) and plots from permutation test (**B**) of normal versus low-metastatic potential cell lines; PLS-DA-derived score plot (**C**) and plots from permutation test (**D**) of normal versus high-metastatic potential cell lines; PLS-DA-derived score plot (**E**) and plots from permutation test (**F**) of low-metastatic potential versus high-metastatic potential cell lines.

As shown in Figure 3, there was clear discrimination between each group: normal cell versus low-metastatic cell lines (Figure 3A), normal cell versus high-metastatic cell lines (Figure 3C), and low-metastatic cell lines versus high-metastatic cell lines (Figure 3E). A permutation test was performed to validate each PLS-DA model. The results of the permutation test with R^2^Y intercept <0.4 and Q^2^Y intercept <0.05 indicated that the model was valid. As shown in Figure 3B,D,F, all PLS-DA models in this study satisfied the criteria of R^2^Y intercept <0.4 and Q^2^Y intercept <0.05 values.

**Figure 4:**
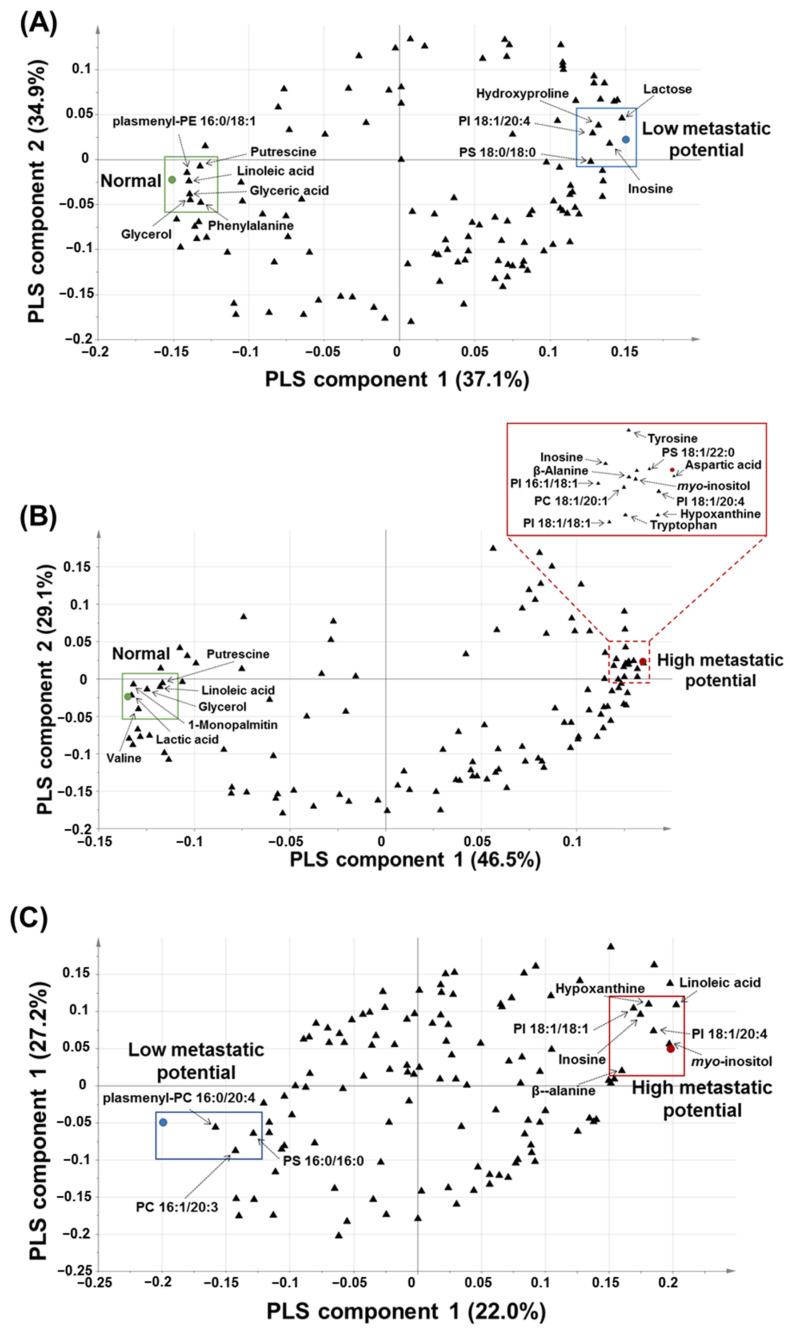
PLS-DA-derived loading plots: (**A**) PLS-DA-derived loading plot of normal versus low-metastatic potential cell lines; (**B**) PLS-DA-derived loading plot of normal versus high-metastatic potential cell lines; (**C**) PLS-DA-derived loading plot of low-metastatic potential versus high-metastatic potential cell lines.

Based on the PLS-DA-derived loading plots and VIP values > 1.0, glyceric acid, glycerol, hydroxyproline, inosine, lactose, linoleic acid, phenylalanine, plasmenyl-phosphatidylethnolamine (PE) 16:0/18:1, phosphatidylinositol (PI) 18:1/20:4, phosphatidylserine (PS) 18:0/18:0, and putrescine were revealed as the major contributing predictive metabolites between the normal cell (HBEpC) and low-metastatic cell lines (H520 and HCC95) (Figure 4A).

β-Alanine, aspartic acid, glycerol, hypoxanthine, inosine, *myo*-inositol, lactic acid, linoleic acid, 1-monopalmitin, phosphatidylcholine (PC) 18:1/20:1, PI 16:1/18:1, PI 18:1/18:1, PI 18:1/20:4, PS 18:1, 22:0, putrescine, tryptophan, tyrosine, and valine were found to be the major contributing metabolites for prediction between the normal cell (HBEpC) and high-metastatic cell lines (SK-MES-1 and H1703) (Figure 4B). 

β-Alanine, hypoxanthine, inosine, linoleic acid, *myo*-inositol, PC 16:1/20:3, PI 18:1/20:4, PI 18:1/18:1, plasmenyl-PC 16:0/22:4, and PS 16:0/16:0 were found to be the major contributing metabolites for prediction between low-metastatic cell lines (H520 and HCC95) and high-metastatic cell lines (SK-MES-1 and H1703) (Figure 4C).

Overall, β-alanine, aspartic acid, glyceric acid, glycerol, hydroxyproline, hypoxanthine, inosine, lactic acid, lactose, linoleic acid, 1-monopalmitin, *myo*-inositol, phenylalanine, PC 18:1/20:1, PI 16:1/18:1, PC 16:1/20:3, PI 18:1/18:1, PI 18:1/20:4, plasmenyl-PE 16:0/18:1, plasmenyl-PC 16:0/22:4, PS 16:0/16:0, PS 18:0/18:0, PS 18:1/22:0, putrescine, tryptophan, tyrosine, and valine were suggested as characteristic metabolites or intact lipid species for the prediction of metastatic potential in the four lung SQCC cell lines. The metabolites, and intact lipid species of VIP values > 1.0 derived from each PLS-DA model were listed in Table S5. Among the characteristic metabolites or intact lipid species, the relative levels of glycerol, putrescine, β-alanine, hypoxanthine, inosine, *myo*-inositol, PI 18:1/18:1, and PI 18:1/20:4 were relatively decreased (glycerol and putrescine) or increased (β-alanine, hypoxanthine, inosine, *myo*-inositol, PI 18:1/18:1, and PI 18:1/20:4) in the four lung SQCC cell lines compared to normal cells. Especially, the relative levels of inosine and PI 18:1/20:4 were differentially observed according to low (H520 and HCC95) and high (SK-MES-1 and H1703) metastatic potentials in lung SQCC. Those two compounds were selected as potential biomarkers to differentiate the lung SQCC according to different metastatic potentials.

### 3.4. Pathway Analysis

**Figure 5:**
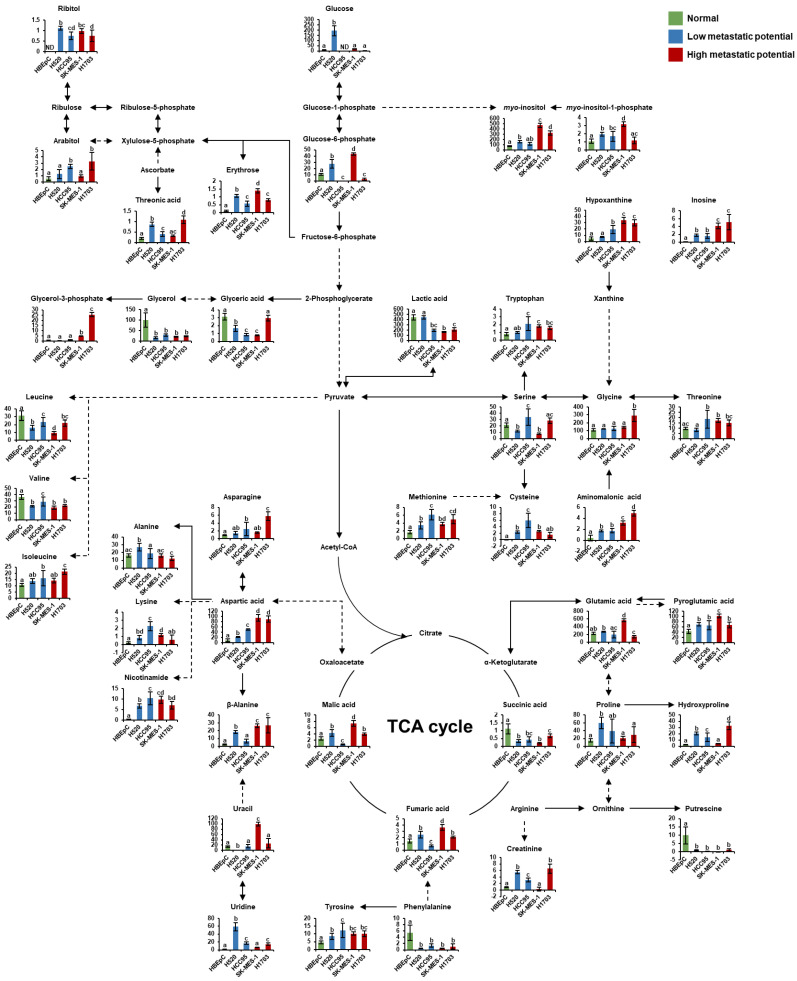
Schematic of metabolism in human bronchial epithelial cells (HBEpC) and four lung squamous cell carcinoma cell lines (H520, HCC95, SK-MES-1, and H1703). The indicated metabolism was modified from the KEGG database (http://www.genome.jp/kegg/ accessed on 5 March 2021). The metabolites on the pathway were selected by associated pathway analysis using Metaboanalyst and each PLS-DA model with a VIP value over 1.0. Each graph shows mean values with error bars indicating standard deviation (SD) (*n* = 8, four biological replicates, and two technical replicates for each group). The *Y*-axis of each data represents the normalized peak intensity. Significant differences were evaluated using ANOVA with a Tukey’s post hoc test, and different letters (a, b, c, and d) represent statistically significant differences of relative levels of each metabolite among samples (*p* < 0.05). Relative levels of each metabolite were represented with different colors according to the cell’s metastatic potential (green, normal cells; blue, low-metastatic potential of lung SQCC; red, high-metastatic potential of lung SQCC). TCA; tricarboxylic acid.

**Figure 6:**
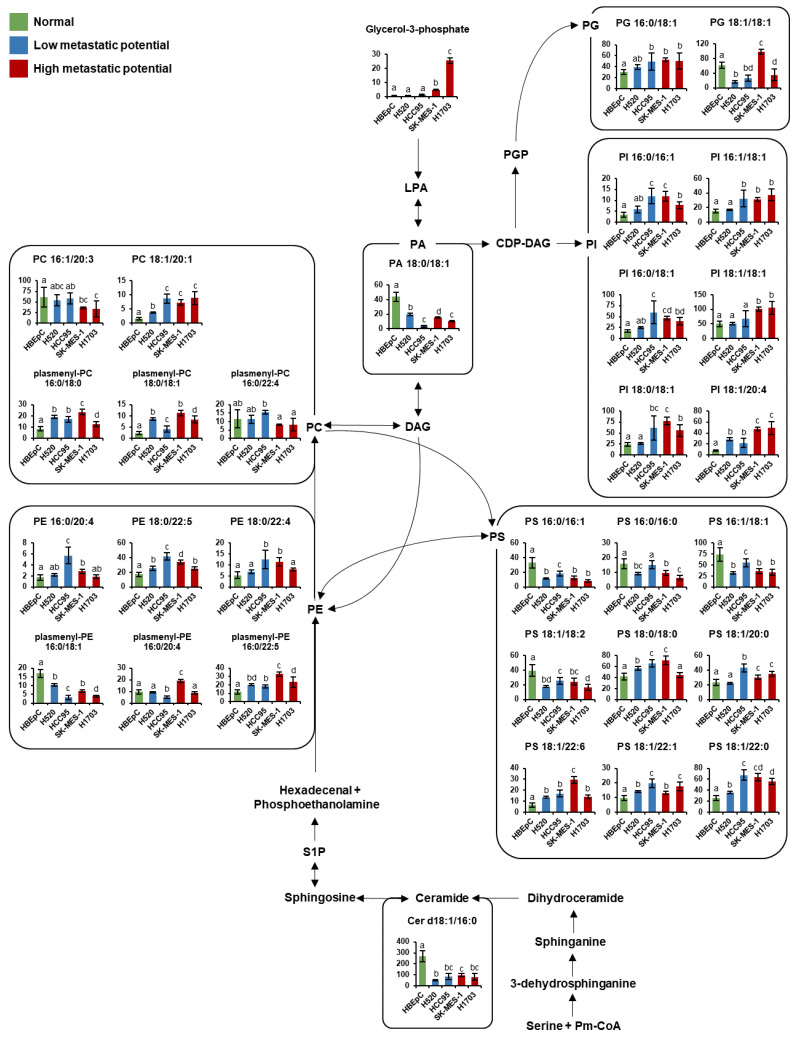
Schematic of lipid pathway in human bronchial epithelial cells (HBEpC) and four lung squamous cell carcinoma cell lines (H520, HCC95, SK-MES-1, and H1703). The indicated lipid pathways were modified from the KEGG database (http://www.genome.jp/kegg/ accessed on 5 March 2021). Indicated lipid species on the pathway were selected by each PLS-DA model with a VIP value over 1.0. Each graph shows mean values with error bars indicating SD (*n* = 8, four biological replicates, and two technical replicates for each group). The *Y*-axis of each data represents the normalized peak intensity. Significant differences were evaluated using ANOVA with a Tukey’s post hoc test and different letters (a, b, c, and d) represent statistically significant differences of relative levels of each intact lipid among samples (*p* < 0.05). Relative levels of each lipid were represented with different colors according to the cell’s metastatic potential (green, normal cells; blue, low-metastatic potential of lung SQCC; red, high-metastatic potential of lung SQCC). Cer, ceramide; CDP-DAG, cytidine diphosphate diacylglycerol; DAG, diacylglycerol; LPA, lysophosphatidic acid; PA, phosphatidic acid; PC, phosphatidylcholine; PE, phosphatidylethanolamine; PG, phosphatidylglycerol; PGP, phosphatidylglycerophosphate; PI, phosphatidylinositol; PS, phosphatidylserine; Pm-CoA; palmitoyl-CoA; S1P, sphingosine-1-phosphate.

To identify key metabolic pathways associated with lung SQCC metastasis, the significantly affected metabolites detected in lung SQCC cell lines were uploaded to MetaboAnalyst [19]. The top eight ranked metabolic pathways with significant cutoffs (*p* < 0.05) and high impact scores were considered to be associated with the metastatic potential of lung SQCC (Table 1). Among them, alanine, aspartate, and glutamate metabolism showed the highest impact value (0.55) and glycine, serine, and threonine metabolism also showed the high impact values (0.42). Arginine and proline metabolism, β-alanine metabolism, aminoacyl-tRNA biosynthesis, glycerolipid metabolism, cysteine, and methionine metabolism, and phenylalanine metabolism were associated with lung SQCC. Figure 5 indicates the major changes of metabolism based on metabolites from pathway analysis and each PLS-DA model with VIP value >1.0. The lipid pathway indicated the glycerophospholipid biosynthesis based on the Kyoto Encyclopedia of Genes and Genomes (KEGG) pathway database, and Figure 6 indicates alteration of lipid pathway based on intact lipid species from each PLS-DA model with VIP value > 1.0. The lipid pathway shows that some PI species indicated an increasing tendency with lung SQCC. In addition, characteristic metabolites and intact lipid species (glycerol and putrescine, β-alanine, hypoxanthine, inosine, *myo*-inositol, PI 18:1/18:1, and PI 18:1/20:4) of lung SQCC metastasis based on the procedure of PLS-DA model including VIP cutoff, and loading plot results were indicated with yellow box (Figure 5 and Figure 6).

## 4. Discussion

In this study, we investigated alterations in the metabolites, intact lipid species, and biological pathways associated with lung SQCC metastasis. In particular, glycerol, putrescine, β-alanine, hypoxanthine, inosine, *myo*-inositol, PI 18:1/18:1, and PI 18:1/20:4 were proposed as characteristic metabolites for the prediction of metastatic potential in lung SQCC. 

In normal cells, glycerol and putrescine showed higher levels, compared with those in lung SQCC cells. Glycerol is converted to glycerol-3-phosphate and glycerol-3-phosphate is essential for cell proliferation and growth [23]. The increased glycerol-3-phosphate level was reported in the lung SQCC tissue, compared to normal tissue [24]. In our study, increased levels of glycerol-3-phosphate were observed in the lung SQCC lines with high-metastatic potential, compared with those in normal cells. The increased conversion of glycerol to glycerol-3-phosphate may induce rapid proliferation in lung SQCC.

Putrescine was a major polyamine of interest in this study; polyamines are known to be necessary for normal and tumor cell proliferation because of their role in the maintenance of oxidative homeostasis, cell membrane structure, and various cellular processes [25,26,27]. The synthesis of polyamines has been shown to be frequently dysregulated in cancer [25]. Increased levels of spermidine and spermine, two products of putrescine, have previously been observed in lung cancer [28,29]. In our study, decreased levels of putrescine in lung SQCC were thought to be due to the increased production of spermidine and spermine.

The high energy demand associated with the rapid proliferation of cancer cells leads to abnormal alterations to numerous metabolic pathways. Our metabolic pathway analysis revealed that alanine, aspartate, and glutamate metabolism, and β-alanine metabolism were associated with lung SQCC. β-Alanine is formed from aspartic acid by GAD1 (glutamic acid decarboxylase 1) and is subsequently metabolized to malonic semialdehyde for fatty acid synthesis or directly converted to pantothenic acid for coenzyme A biosynthesis [30,31]. In human oral squamous cell carcinoma and lung AC, the GAD 1 overexpression was established and revealed that related to invasiveness and metastasis [32,33]. In our results, significantly increased levels of aspartic acid and β-alanine were observed in lung SQCC. Aspartic acid was reported to have the role of transferring electrons between the cytosol and mitochondria for cell proliferation and is essential for purine and pyrimidine synthesis [34,35]. In cancer cells, the addition of aspartic acid showed an increased proliferation rate of cancer cells such as melanoma cells, lung cancer cells, cervical cancer cells, and glioblastoma cells, even though it inhibited electron transport chain function [36]. Therefore, the elevated β-alanine levels in lung SQCC may have resulted from increased aspartic acid and GAD1 levels, contributing to activation of energy production for metastasis.

As building blocks for DNA and RNA, purine derivatives are responsible for nucleic acid synthesis and energy production. Our study revealed that hypoxanthine and inosine concentrations were increased with increasing metastatic potential. In the previous study, higher levels of hypoxanthine and inosine were observed in lung SQCC tissue, compared to normal tissue [24]. Significantly higher levels of adenosine triphosphate (ATP) and adenosine diphosphate (ADP) were suggested as characteristic of lung SQCC tissues versus lung adenocarcinoma and large cell carcinoma [37]. The accumulation of those purine derivatives might be characteristics of lung SQCC, compared to other types of lung cancer. In addition, we observed higher cell proliferation in low-metastatic (H520 and HCC95) and high-metastatic (H1703) cell lines, compared to normal cell lines (HBEpC) by BrdU cell proliferation assay. Higher levels of hypoxanthine and inosine in high-metastatic (H1703) cell lines might contribute to the increase of DNA synthesis resulted in an increase in cell proliferation. However, the lowest cell proliferation was observed in the SK-MES-1 cell line. There was no correlation between the higher levels of hypoxanthine/inosine and cell proliferation in the SK-MES-1 cell line.

An important factor to shape characteristics of cancer metastasis is cell motility such as invasion and migration [38]. The upregulation of vimentin and downregulation of E-cadherin expression are indicators of cancer invasiveness and metastasis [20,21]. In our study, high-metastatic (H1703 and SK-MES-1) cell lines showed higher expression of vimentin and loss of E-cadherin, compared to low-metastatic cell lines. In colorectal cancer cells, knockdown of phosphoribosylaminoimidazole carboxylase, phosphoribosylaminoimidazole succinocarboxamide synthetase (PAICS) induced higher levels of E-cadherin and lower levels of vimentin [39]. PAICS, an de novo purine biosynthesis enzyme, was overexpressed in lung SQCC, breast cancer, and colorectal cancer cells [39,40,41]. It is implied that enhanced purine biosynthesis is characteristics of cancer cells and their metastasis. In normal cells, hypoxanthine and inosine are metabolized to uric acid by xanthine oxo-reductase through purine metabolism. However, cancer cells, which require a high rate of DNA synthesis, activate de novo purine synthesis by disrupting purine metabolism via a decrease in xanthin oxo-reductase [42,43,44]. Thus, the increased levels of hypoxanthine and inosine in lung SQCC cells could be potentially due to the alteration of purine metabolism to enable the rapid proliferation and metastasis of lung SQCC. Particularly, the relative level of inosine would be considered a potential biomarker for estimating lung SQCC metastasis due to the subsequently increasing level according to different metastatic potentials of lung SQCC.

The lipid pathway analysis revealed that some PI species showed an increased level of tendency correlated with lung SQCC. Particularly PI 18:1/18:1 and PI 18:1/20:4, which showed the highest levels in high-metastatic cell lines. There was a report that the increased levels of PI species in SQCC tissue, compared to normal lung tissue from patients [45]. In addition, significantly higher levels of PI species were reported in lung SQCC compared to lung adenocarcinoma tissue [46]. PI species are known as the precursors of phosphatidylinositol-3,4,5-trisphosphate (PIP3), which regulates cell growth, proliferation, and migration [47,48]. PIP3 is synthesized by phosphatidylinositol-3-kinase (PI3K), and it serves as a docking site to recruit Akt to the cellular membrane, where Akt is activated by PDK1 and PDK2 [49]. It is also known that PI3K/Akt signaling regulates tumor growth and metastasis [50,51]. We observed higher expression of *p*-AKT in high-metastatic cell lines (H1703). Lower expression of *p*-AKT in SK-MES-1 might be due to activating other pathways such as SGK, S6K, and PKC pathway through PI3K signaling [52]. Our results revealed that the levels of most PI species were increased in lung SQCC samples, compared with those in HBEpC. In particular, PI 18:1/18:1 and PI 18:1/20:4, together with *myo*-inositol (a precursor to PI), were significantly increased in cell lines with high-metastatic potential. It may imply the increased PI species enhance the lung SQCC growth and metastasis. In particular, the distinct difference level of PI 18:1/20:4 was observed according to different metastatic potentials in lung SQCC. Therefore, it is suggested that increased PI species induced enhanced metastasis of SQCC cells via PI3K/Akt signaling.

Altered gene expression is closely related to cancer metabolism including lipid metabolism [53]. There was a report that glycerophospholipid metabolism, fatty acid metabolism, and eicosanoid signaling were significantly enriched in lung adenocarcinoma compared to normal tissue [54]. In addition, the MYC gene has been known as an oncogene that promotes cancer cell growth and proliferation [55]. In a previous study, PI 18:0/18:2 and PI 18:0/20:3 were mostly increased in MYC gene-activated lymphoma, compared to inactivated lymphoma [56]. PC levels were also increased in MYC-activated lung tumor tissue, compared to inactivated tumor tissue [57]. It is implied the MYC gene expression would regulate the levels of glycerophospholipids. Overexpression of the MYC gene was also observed in lung SQCC tissue, compared to lung adenocarcinoma tissue [58]. PI species increased in the lung SQCC cells, compared to normal cells in our study. Thus, it is speculated that increased levels of PI species in lung SQCC might be controlled by the MYC gene.

We performed an integrated comprehensive metabolite and intact lipid species profiling analysis to reveal the major metastasis-related metabolites and intact lipid species in lung SQCC. This study constructed validated PLS-DA models of characteristic metabolites and intact lipid species to predict the metastatic potential of lung SQCC and identified therapeutic targets for the inhibition of lung SQCC metastasis. Our approach provides meaningful insights into lung SQCC metastasis-related biological pathways.

## 5. Conclusions

This study aimed to develop the prediction model for lung SQCC metastasis and identify therapeutic targets for the inhibition of lung SQCC metastasis. This is the first report of integrated comprehensive metabolomic and lipidomic profiling in the lung SQCC according to different metastatic potentials. Since this study employed in vitro samples, the proposed therapeutic targets need to be validated by in vivo and clinical studies in the future.

## Figures and Tables

**Table 1:** Key metabolic pathways associated with lung squamous cell cancer metastasis.

No.	Metabolism	Interaction Metabolite	Total ^1^	Hits ^2^	*p* ^3^	Impact ^4^
1	Alanine, aspartate and glutamate metabolism	Alanine, asparagine, aspartic acid, fumaric acid, glutamic acid, succicnic acid	24	6	7.58 × 10^−6^	0.55
2	Glycine, serine, and threonine metabolism	Aspartic acid, cysteine, glycine, glyceric acid, serine, threonine, tryptophan	48	7	5.42 × 10^−5^	0.42
3	Arginine and proline metabolism	Aspartic acid, creatinine, fumaric acid, glutamic acid, hydroxyproline, proline, putrescine,	77	7	1.09 × 10^−3^	0.27
4	β-Alanine metabolism	β-Alanine, aspartic acid, uracil	28	3	2.13 × 10^−2^	0.26
5	Aminoacyl-tRNA biosynthesis	Alanine, asparagine, aspartic acid, cysteine, glutamic acid, glycine, isoleucine, leucine, lysine, methionine, serine phenylalanine, proline, threonine, tryptophan, tyrosine, valine	75	17	3.55 × 10^−14^	0.23
6	Glycerolipid metabolism	Glyceric acid, glycerol, glycerol-3-phosphate	32	3	3.04 × 10^−2^	0.22
7	Cysteine and methionine metabolism	Alanine, asparagine, cysteine, methionine, serine	56	5	6.42 × 10^−3^	0.18
8	Phenylalanine metabolism	Fumaric acid, phenylalanine, succinic acid, tyrosine	45	4	1.50 × 10^−2^	0.12

^1^ Total is the total number of compounds in the metabolism. ^2^ Hits is the matched number of compounds from the uploaded data set. ^3^ *p* is the *p* value calculated from the pathway analysis. ^4^ Impact is the cumulative percentage from matched metabolites in each total pathway. The threshold of 0.1 was set to filter less important pathways.

## Data Availability

The data presented in this study are available on reasonable request from the corresponding author.

## Supplementary Materials

The following are available online at https://www.mdpi.com/article/10.3390/cancers13164179/s1, Figure S1: Relative mRNA level of vimentin and E-cadherin in four lung SQCC cell lines, Figure S2: Cell proliferation of human bronchial epithelial cells (HBEpC) and four lung SQCC cell lines, Figure S3: (A) Expression of AKT and p-AKT of four lung SQCC cell lines; (B) Uncropped blot image for each antibody (AKT, p-AKT, and actin), Figure S4: Uncropped blot image for each antibody (vimentin, E-cadherin, and actin) related to Figure 1, Figure S5: PCA-derived score plot of HBEpC and four lung SQCC, Table S1: List of primer sequences for RT-PCR analysis, Table S2: Identification and relative levels of metabolites in HBEpC and lung SQCC cell lines (H520, HCC95, SK-MES-1, and H1703) analyzed by GC-MS, Table S3: Lipid species identified from HBEpC and lung SQCC cells by DI-MS analysis, Table S4: Relative levels of identified lipids in HBEpC and lung SQCC cell lines (H520, HCC95, SK-MES-1, and H1703) analyzed by DI-MS, Table S5: Metabolites and intact lipid species with variable influence on projection (VIP) values higher than 1.0.
