# Supplementary material for: Integrative Metabolomic and Lipidomic Profiling of Lung Squamous Cell Carcinoma for Characterization of Metabolites and Intact Lipid Species Related to the Metastatic Potential
Source: Cancers (Basel). 2021 Aug 19;13(16):4179. doi: 10.3390/cancers13164179 (PMC8391613; doi:10.3390/cancers13164179)
Supplement: Supplementary file 1 [file cancers-13-04179-s001.zip › cancers-1230752-supplementary.pdf]

Supplementary Material

Integrative metabolomic and lipidomic profiling of lung squamous cell carcinoma for characterization of metabolites and intact lipid species related to the metastatic potential

Heayyean Lee, Hwanhui Lee, Sujeong Park, Myeongsun Kim, Ji Young Park, Hanyong Jin, Kyungsoo Oh, Jeehyeon Bae, Young Yang, Hyung-Kyoon Choi

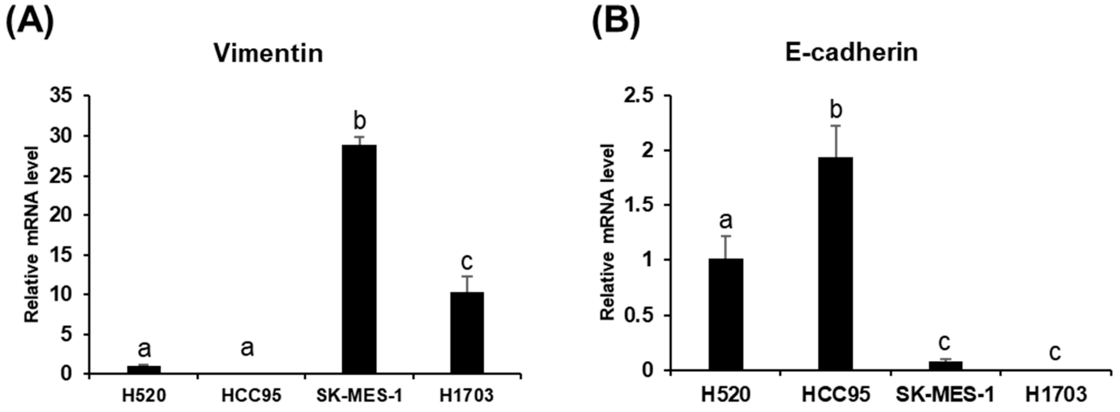

Figure S1. Relative mRNA level of vimentin and e-cadherin in four lung SQCC cell lines. Significant differences were evaluated using ANOVA with a Tukey's post-hoc test and different letters represent statistically significant differences among samples ($p < 0.05$).

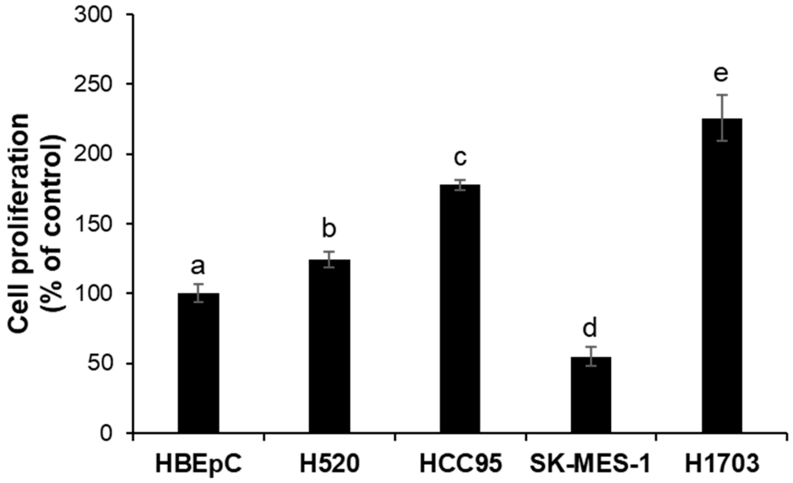

Figure S2. Cell proliferation of human bronchial epithelial cells (HBEpC) and four lung SQCC cell lines. HBEpC cells were used as a control. The cell proliferation assay is based on the incorporation of BrdU as a marker of DNA synthesis. Significant differences were evaluated using ANOVA with a Tukey's post-hoc test and different letters represent statistically significant differences among samples ($p < 0.05$).

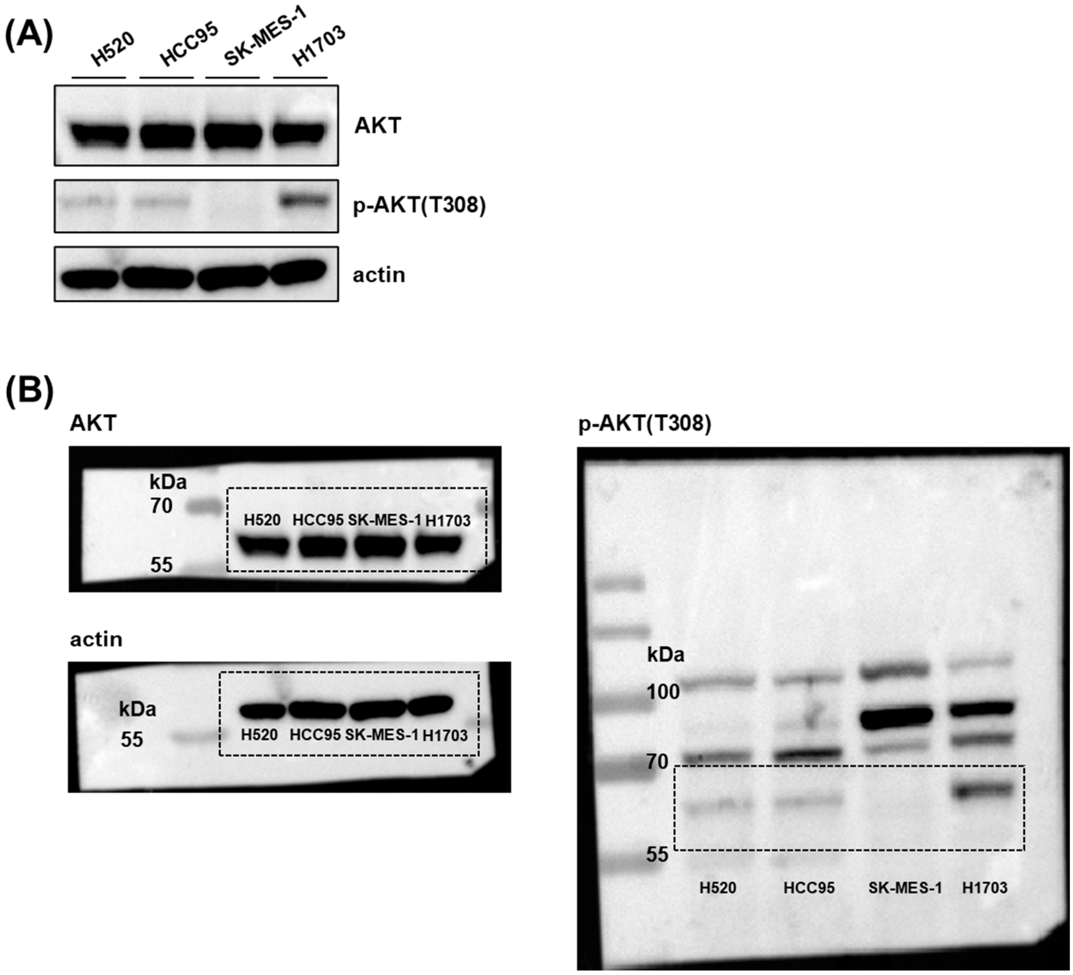

Figure S3. (A) Expression of AKT and p-AKT of four lung SQCC cell lines; (B) Uncropped blot image for each antibody (AKT, p-AKT, and actin). The actin was used as a loading control.

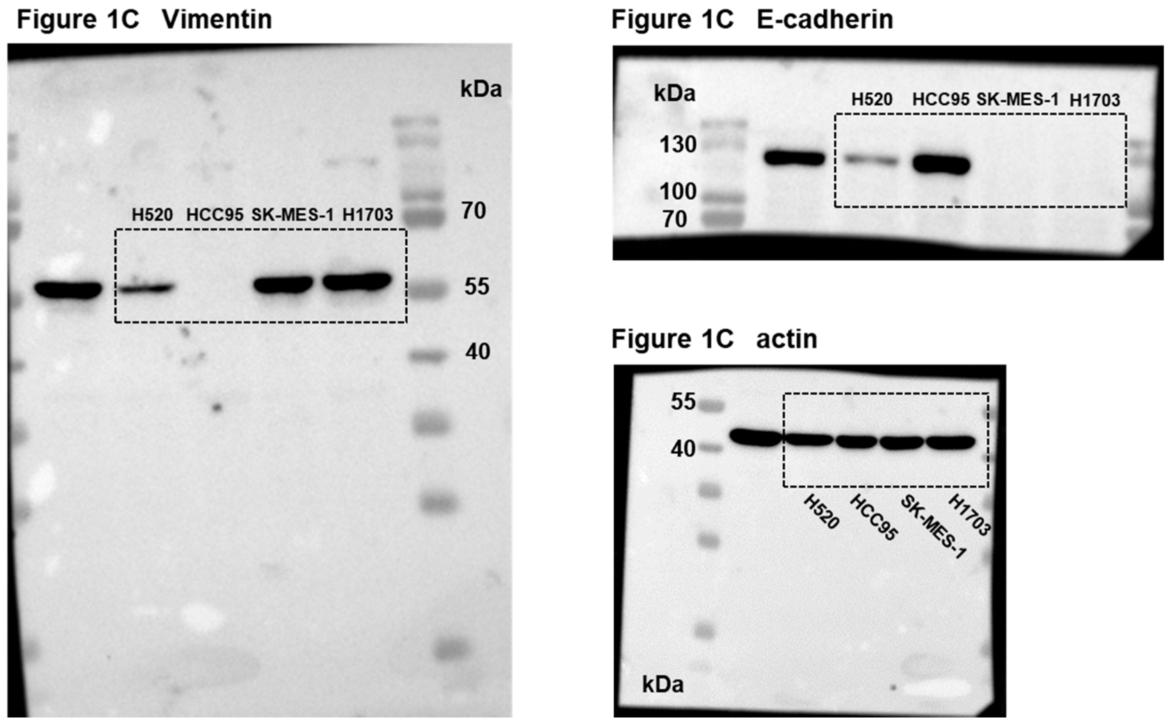

Figure S4. Uncropped blot image for each antibody (vimentin, E-cadherin, and actin) related to Figure 1. The actin was used as a loading control.

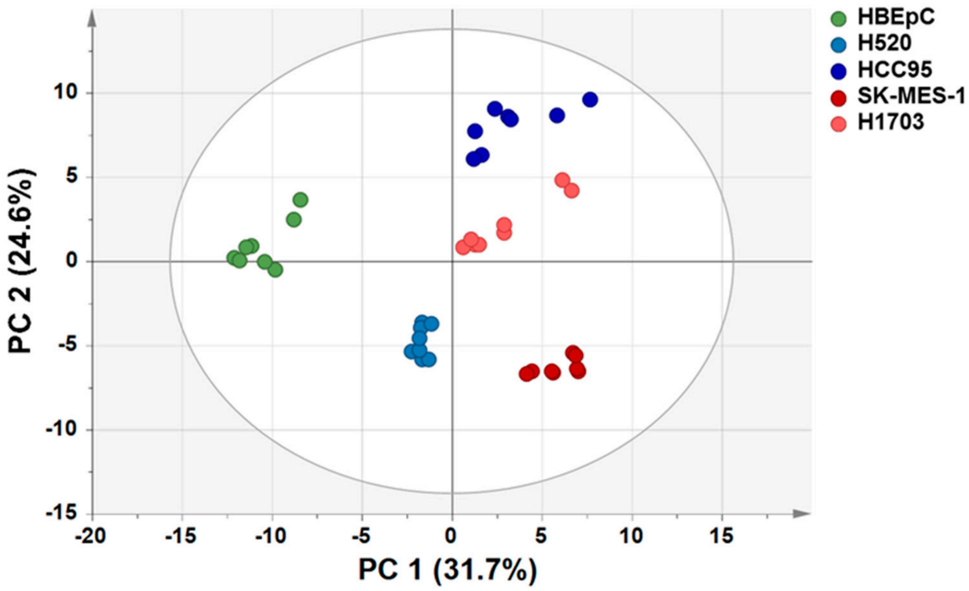

Figure S5. PCA-derived score plot of HBEpC and four lung SQCC. PCA-derived score plot based on metabolic and lipidomic data from HBEpC and four lung SQCC (H520, HCC95, SK-MES-1, and H1703), which exhibited different metastatic potentials (n = 8, four biological replicates and two technical replicates for each group). The score plot was obtained after unit variance scaling pretreatment.

Table S1. List of primer sequences for RT-PCR analysis.

	Primer sequence	
**Gene**	**Sequence (forward)**	**Sequence (reverse)**
Vimentin	5′- GAGAACTTTGCCGTTGAAGC-3′	5′- TCCAGCAGCTTCCTGTAGGT-3′
E-cadherin	5′-CAATGCCGCCATCGCTTA C-3′	5′- ATGACTCCTGTGTTC CTGTTAATG-3′
GAPDH	5′-ACCCAGAAGACTGTGGATGG-3′	5′-TCTAGACGGCAGGTCAGGTC-3′

Table S2. Identification and relative levels of metabolites in HBEpC and lung SQCC cell lines (H520, HCC95, SK-MES-1, and H1703) analyzed by GC-MS.

No	Compound	m/z	RT	Mass fragmentation	TMS	Normal	Low metastatic potential		High metastatic potential	
						HBEpC	H520	HCC95	SK-MES-1	H1703
	**Amino acids and derivates**									
1	Alanine	116	6.98	100, **116**, 190, 218	2	16.43 ± 2.24[ac]	26.83 ± 4.67[b]	18.76 ± 6.30[a]	16.17 ± 2.11[ac]	12.52 ± 2.72[c]
2	β-Alanine	174	15.08	133, **174**, 248, 290	3	2.57 ± 0.72[a]	18.59 ± 1.82[b]	6.99 ± 2.45[a]	26.10 ± 2.52[c]	26.82 ± 9.54[c]
3	Aminomalonic acid	218	16.11	133, 174, **218**, 320	3	0.37 ± 0.51[a]	1.69 ± 0.20[b]	1.74 ± 0.43[b]	3.15 ± 0.39[c]	4.91 ± 0.50[d]
4	Asparagine	159	19.18	100, 116, 130, **159**	2	0.89 ± 0.10[a]	1.43 ± 0.47[ab]	2.50 ± 1.68[b]	1.57 ± 0.22[ab]	5.76 ± 1.11[c]
		116	20.80	**116**, 132, 188, 231	3					
5	Aspartic acid	160	15.04	117, 130, **160**, 245	2	8.48 ± 5.11[a]	21.29 ± 1.91[b]	49.97 ± 3.30[c]	94.78 ± 12.77[d]	89.82 ± 11.68[d]
		232	17.38	100, 202, 218, **232**	3					
6	Cysteine	220	18.16	100, **220**, 294, 322	3	0.01 ± 0.03[a]	2.41 ± 0.38[b]	5.98 ± 2.19[c]	2.60 ± 0.24[b]	1.48 ± 0.76[ab]
7	Glutamic acid	246	19.72	128, 156, **246**, 348	3	229.29 ± 27.37[ab]	273.64 ± 11.63[b]	198.34 ± 85.60[ac]	573.51 ± 36.88[d]	145.69 ± 28.25[c]
8	Glycine	102	7.45	**102**, 131, 176, 204	2	110.96 ± 16.47[a]	121.51 ± 3.69[a]	127.65 ± 28.09[a]	142.62 ± 11.62[a]	293.40 ± 73.52[b]
		174	11.99	86, **174**, 248, 276	3					
9	Hydroxyproline	230	17.48	140, **230**, 304, 332	3	2.18 ± 0.43[a]	20.39 ± 2.54[b]	14.34 ± 6.68[c]	4.41 ± 0.29[a]	32.72 ± 6.11[d]
10	Isoleucine	86	8.80	75, **86**, 146, 188	1	10.79 ± 1.22[a]	13.92 ± 1.97[ab]	16.22 ± 6.20[b]	14.25 ± 1.96[ab]	21.31 ± 2.25[c]
		158	11.68	100, **158**, 218, 232	2					

11	Leucine	86	8.28	75, **86**, 146, 188	1	31.18 ± 6.06[a]	16.10 ± 2.83[b]	23.51 ± 5.58[c]	9.08 ± 2.02[d]	21.95 ± 3.82[bc]
12	Lysine	174	26.02	156, **174**, 230, 317	4	0.21 ± 0.12[a]	0.84 ± 0.25[bd]	2.29 ± 0.57[c]	1.18 ± 0.16[d]	0.57 ± 0.59[ab]
13	Methionine	104	14.71	61, **104**, 131, 221	1	1.69 ± 0.44[a]	3.42 ± 0.91[b]	6.13 ± 1.37[c]	3.74 ± 0.43[bd]	4.97 ± 1.13[cd]
		176	17.29	61, 128, **176**, 219	2					
14	Phenylalanine	120	17.97	75, 91, **120**, 146	1	5.41 ± 2.41[a]	0.42 ± 0.10[b]	1.35 ± 0.48[b]	0.44 ± 0.07[b]	1.05 ± 0.81[b]
15	Proline	70	8.75	**70**, 75, 103, 172	1	15.40 ± 4.05[a]	59.49 ± 14.50[b]	39.33 ± 30.61[ab]	21.22 ± 4.77[a]	29.99 ± 20.17[a]
		142	11.77	100, **142**, 216, 244	2					
16	Pyroglutamic acid	156	17.32	**156**, 230, 258, 273	2	42.64 ± 8.96[a]	69.85 ± 5.91[b]	66.27 ± 17.27[b]	101.58 ± 7.24[c]	66.51 ± 10.93[b]
17	Serine	116	10.78	103, **116**, 132, 219	2	21.81 ± 3.40[a]	11.92 ± 1.71[b]	34.10 ± 12.97[c]	7.60 ± 1.05[b]	28.42 ± 4.43[ac]
		204	13.41	100, **204**, 218, 278	3					
18	Threonine	117	11.70	**117**, 130, 158, 219	2	9.69 ± 1.31[ac]	8.40 ± 1.62[a]	18.46 ± 8.43[b]	17.08 ± 1.79[b]	15.01 ± 2.74[bc]
		218	14.03	101, 117, **218**, 278	3					
19	Tryptophan	202	30.70	100, 130, **202**, 218	2	0.81 ± 0.22[a]	1.00 ± 0.12[ab]	2.12 ± 0.89[c]	1.82 ± 0.16[c]	1.59 ± 0.21[bc]
20	Tyrosine	179	25.32	**179**, 208, 219, 310	2	4.68 ± 0.89[a]	8.45 ± 2.06[b]	12.26 ± 4.47[c]	10.24 ± 1.17[bc]	10.08 ± 1.74[bc]
		218	26.32	100, 179, **218**, 280	3					
21	Valine	72	6.71	55, **72**, 146, 174	1	36.34 ± 4.01[a]	21.46 ± 1.07[b]	28.88 ± 7.07[c]	19.15 ± 2.54[b]	22.23 ± 1.59[b]
		144	9.70	100, **144**, 218, 246	2					

	**Fatty acids**								
22	1-Monopalmitin	371	36.35	205, 239, **371**, 459	2	155.74 ± 17.43[a]	91.69 ± 21.51[b]	91.83 ± 21.32[b]	61.62 ± 9.08[c]	78.06 ± 10.28[bc]
23	11-Eicosenoic acid	367	33.93	117, 129, 145, **367**	1	0.07 ± 0.13[a]	0.84 ± 0.09[b]	2.01 ± 0.56[c]	0.85 ± 0.06[b]	1.29 ± 0.22[d]
24	Arachidonic acid	75	33.21	**75**, 91, 117, 129	1	0.51 ± 0.55[a]	1.71 ± 0.11[b]	1.70 ± 0.32[b]	2.47 ± 0.16[c]	1.56 ± 0.16[b]
25	Linoleic acid	337	30.93	95, 117, 129, **337**	1	1.55 ± 0.57[a]	0.28 ± 0.03[b]	0.25 ± 0.05[b]	0.48 ± 0.03[b]	0.52 ± 0.07[b]
26	Oleic acid	339	31.04	96, 117, 129, **339**	1	76.08 ± 28.45[a]	32.19 ± 3.05[b]	51.60 ± 10.09[b]	37.67 ± 1.69[b]	45.79 ± 5.28[b]
27	Palmitic acid	313	28.31	117, 132, 145, **313**	1	64.17 ± 21.64[a]	37.63 ± 4.72[b]	41.98 ± 7.62[b]	35.60 ± 1.15[b]	33.65 ± 2.26[b]
28	Stearic acid	341	31.45	117, 132, 145, **341**	1	27.24 ± 7.32[ab]	30.95 ± 2.92[b]	23.71 ± 4.12[ac]	47.57 ± 1.52[d]	18.69 ± 1.35[c]
	**Miscellaneous metabolites**									
29	Creatinine	115	18.04	100, **115**, 314, 329	3	0.90 ± 0.18[a]	5.47 ± 0.52[b]	3.04 ± 0.58[c]	0.29 ± 0.55[a]	6.56 ± 1.42[b]
30	Nicotinamide	179	16.42	75, 105, 136, **179**	1	0.27 ± 0.06[a]	6.67 ± 1.10[b]	10.35 ± 3.09[c]	9.65 ± 1.66[cd]	7.09 ± 1.80[bd]
31	Phosphoric acid	299	11.15	133, 211, **299**, 314	3	2387.32 ± 322.48[a]	2528.50 ± 485.01[a]	2260.26 ± 196.23[a]	2602.82 ± 134.71[a]	2683.33 ± 328.93[a]
32	Putrescine	174	22.24	100, **174**, 200, 214	4	9.87 ± 5.18[a]	0.91 ± 0.30[b]	0.08 ± 0.15[b]	0.05 ± 0.05[b]	1.15 ± 0.75[b]
	**Organic acids**									
33	Fumaric acid	245	13.22	75, 115, 133, **245**	2	1.51 ± 0.30[a]	2.47 ± 0.53[b]	0.71 ± 0.16[c]	3.61 ± 0.44[d]	2.08 ± 0.14[b]
34	Lactic acid	117	6.04	**117**, 133, 191, 219	2	446.19 ± 50.32[a]	443.31 ± 33.18[a]	196.73 ± 23.61[bc]	162.06 ± 10.37[b]	211.53 ± 30.99[c]

35	Malic acid	233	16.62	133, **233**, 245, 335	3	2.51 ± 0.51[a]	4.31 ± 1.08[b]	0.61 ± 0.15[c]	7.33 ± 0.88[d]	3.93 ± 0.38[b]
36	Succinic acid	247	12.27	129, 172, 218, 247	2	1.13 ± 0.32[a]	0.34 ± 0.08[b]	0.44 ± 0.17[bc]	0.21 ± 0.04[b]	0.68 ± 0.13[c]
	**Purines and pyrimidines**									
37	Hypoxanthine	265	23.70	193, 206, **265**, 280	2	4.73 ± 2.92[a]	7.17 ± 0.81[a]	19.24 ± 6.27[b]	33.85 ± 4.94[c]	29.74 ± 4.81[c]
38	Inosine	217	36.07	**217**, 230, 245, 281	4	0.11 ± 0.08[a]	1.81 ± 0.28[b]	1.58 ± 0.62[b]	4.11 ± 0.73[c]	5.15 ± 1.98[c]
39	Uracil	241	12.81	99, 113, **241**, 256	3	14.91 ± 2.33[a]	0.24 ± 0.09[b]	15.84 ± 6.28[a]	99.13 ± 7.57[c]	26.95 ± 16.95[a]
40	Uridine	217	34.28	103, 169, **217**, 259	3	1.62 ± 0.45[a]	59.00 ± 10.33[b]	16.72 ± 3.04[c]	6.22 ± 0.71[a]	14.49 ± 3.13[c]
	**Carbohydrates and related compounds**									
41	Arabitol	217	21.86	103, 205, **217**, 307	5	0.54 ± 0.28[a]	1.26 ± 0.75[a]	2.48 ± 0.35[b]	0.93 ± 0.18[a]	3.30 ± 1.39[b]
42	Erythrose	217	17.06	103, 205, **217**, 307	3	0.11 ± 0.05[a]	1.07 ± 0.09[b]	0.57 ± 0.16[c]	1.39 ± 0.15[d]	0.81 ± 0.09[e]
43	Glucose	204	27.10	129, 191, **204**, 217,	5	9.12 ± 1.70[a]	194.00 ± 47.99[b]	ND	19.23 ± 1.35[a]	0.54 ± 0.44[a]
44	Glucose-6-phosphate	387	33.28	129, 204, 299, **387**	6	11.20 ± 1.48[a]	27.57 ± 6.94[b]	0.30 ± 0.28[c]	43.50 ± 1.83[d]	3.22 ± 2.03[c]
45	Lactose	204	36.95	191, **204**, 217, 361	8	ND	2.87 ± 0.47[b]	2.93 ± 0.75[b]	2.15 ± 0.23[b]	2.17 ± 1.13[b]
46	Mannose-6-phosphate	204	31.86	129, **204**, 299, 387	6	0.65 ± 0.10[a]	0.99 ± 0.22[b]	0.01 ± 0.02[c]	1.30 ± 0.05[d]	0.15 ± 0.12[c]
47	Glyceric acid	189	12.63	103, 133, **189**, 292	3	3.13 ± 0.43[a]	1.65 ± 0.40[b]	0.85 ± 0.15[c]	0.78 ± 0.07[c]	2.97 ± 0.38[a]
48	Glycerol	205	11.21	103, 117, **205**, 218	3	101.23 ± 34.46[a]	16.33 ± 4.24[b]	29.00 ± 5.84[b]	21.13 ± 2.40[b]	22.28 ± 2.48[b]
49	Glycerol-3-phosphate	357	22.82	299, 315, **357**, 445	4	0.67 ± 0.43[a]	0.62 ± 0.10[a]	1.28 ± 0.33[a]	4.80 ± 0.25[b]	25.47 ± 1.99[c]
50	*myo*-inositol	305	28.90	191, 217, **305**, 318	6	75.03 ± 8.78[a]	157.86 ± 19.85[b]	114.12 ± 26.42[ab]	469.69 ± 41.97[c]	324.25 ± 41.92[d]

51	*myo*-inositol-1-phosphate	318	33.75	299, 315, **318**, 387	7	1.06 ± 0.27^{a}	1.95 ± 0.24^{b}	1.68 ± 0.61^{bc}	3.18 ± 0.28^{d}	1.17 ± 0.41^{ac}
52	Ribitol	217	21.96	103, 205, **217**, 319	5	ND	1.12 ± 0.10^{b}	0.76 ± 0.19^{cd}	0.99 ± 0.12^{bc}	0.75 ± 0.27^{d}
53	Ribose	217	20.38	103, 191, 204, **217**	4	1.57 ± 0.12^{a}	4.66 ± 0.70^{b}	1.50 ± 0.26^{a}	4.97 ± 0.39^{b}	3.54 ± 0.38^{c}
		103	20.96	**103**, 205, 217, 307	4 (MeOX)					
54	Threonic acid	292	17.88	117, 205, 220, **292**	4	0.20 ± 0.05^{a}	0.87 ± 0.10^{b}	0.40 ± 0.11^{c}	0.32 ± 0.03^{ac}	1.09 ± 0.18^{d}

*ND, not detected; RT, retention time; TMS, trimethylsilylation.

Each value represents the mean ± SD (n = 8). Significant differences were evaluated using ANOVA with a Tukey's *post-hoc* test and different letters represent statistically significant differences among samples ($p < 0.05$).

Table S3. Lipid species identified from HBEpC and lung SQCC cell lines by DI-MS analysis.

No.	Lipid species	Ion species	m/z	MS/MS fragment ion (m/z)
	**Positive ion mode**			
	Phosphatidylcholine (PC)			
1	PC 16:0/16:1	$[M + H]^+$	732	**476** [lyso-PC(16:1) – H_2O + H]$^+$; **478** [lyso-PC(16:0) – H_2O + H]$^+$; **494** [lyso-PC(16:1) + H]$^+$; **496** [lyso-PC(16:0) + H]$^+$; **549** [M – $C_5H_{14}NO_4P$ + H]$^+$; **673** [M – C_3H_9N + H]$^+$; **714** [M – H_2O + H]$^+$
2	PC 16:0/16:0	$[M + H]^+$	734	**478** [lyso-PC(16:0) – H_2O + H]$^+$; **496** [lyso-PC(16:0) + H]$^+$; **551** [M – $C_5H_{14}NO_4P$ + H]$^+$; **675** [M – C_3H_9N + H]$^+$; **716** [M – H_2O + H]$^+$
3	PC 16:1/18:1	$[M + H]^+$	758	**476** [lyso-PC(16:1) – H_2O + H]$^+$; **494** [lyso-PC(16:1) + H]$^+$; **504** [lyso-PC(18:1) – H_2O + H]$^+$; **522** [lyso-PC(18:1) + H]$^+$; **575** [M – $C_5H_{14}NO_4P$ + H]$^+$; **699** [M – C_3H_9N + H]$^+$; **740** [M – H_2O + H]$^+$
4	PC 16:0/18:1	$[M + H]^+$	760	**478** [lyso-PC(16:0) – H_2O + H]$^+$; **496** [lyso-PC(16:0) + H]$^+$; **504** [lyso-PC(18:1) – H_2O + H]$^+$; **522** [lyso-PC(18:1) + H]$^+$; **577** [M – $C_5H_{14}NO_4P$ + H]$^+$; **701** [M – C_3H_9N + H]$^+$; **742** [M – H_2O + H]$^+$
5	PC 16:0/18:0	$[M + H]^+$	762	**478** [lyso-PC(16:0) – H_2O + H]$^+$; **496** [lyso-PC(16:0) + H]$^+$; **506** [lyso-PC(18:0) – H_2O + H]$^+$; **524** [lyso-PC(18:0) + H]$^+$; **579** [M – $C_5H_{14}NO_4P$ + H]$^+$; **703** [M – C_3H_9N + H]$^+$; **744** [M – H_2O + H]$^+$
6	PC 18:1/18:1	$[M + H]^+$	786	**504** [lyso-PC(18:1) – H_2O + H]$^+$; **522** [lyso-PC(18:1) + H]$^+$; **603** [M – $C_5H_{14}NO_4P$ + H]$^+$; **727** [M – C_3H_9N + H]$^+$; **768** [M – H_2O + H]$^+$
7	PC 18:0/18:1	$[M + H]^+$	788	**504** [lyso-PC(18:1) – H_2O + H]$^+$; **506** [lyso-PC(18:0) – H_2O + H]$^+$; **522** [lyso-PC(18:1) + H]$^+$; **524** [lyso-PC(18:0) + H]$^+$; **605** [M – $C_5H_{14}NO_4P$ + H]$^+$; **729** [M – C_3H_9N + H]$^+$; **770** [M – H_2O + H]$^+$
8	PC 16:1/20:3	$[M + Na]^+$	804	**439** [(M + Na) – C_3H_9N – C20:3]$^+$; **491** [(M + Na) – C_3H_9N – C16:0]$^+$; **516** [(M + Na) – C20:3 + H_2O]$^+$; **550** [(M + Na) – C16:1]+; **621** [(M + Na) – $C_5H_{14}NO_4P$]$^+$; **745** [(M + Na) – C_3H_9N]$^+$
9	PC 18:0/18:0	$[M + Na]^+$	812	**469** [(M + Na) – C_3H_9N – C18:0]$^+$; **528** [(M + Na) – H_2O – C18:0]$^+$; **629** [(M + Na) – $C_5H_{14}NO_4P$]$^+$; **753** [(M + Na) – C_3H_9N]$^+$
10	PC 18:0/20:4	$[M + Na]^+$	832	**469** [(M + Na) – C_3H_9N – C20:4]$^+$; **489** [(M + Na) – C_3H_9N – C18:0]$^+$; **528** [(M + Na) – C20:4]$^+$; **546** [(M + Na) – C20:4 + H_2O]$^+$; **548** [(M + Na) – C18:0]$^+$; **566** [(M + Na) – C18:0 + H_2O]$^+$; **649** [(M + Na) – $C_5H_{14}NO_4P$]$^+$; **773** [(M + Na) – C_3H_9N]$^+$

11	PC 18:1/20:2	$[M + Na]^+$	834	**467** $[(M + Na) – C_3H_9N – C20:4]^+$; **493** $[(M + Na) – C_3H_9N – C18:0]^+$; **526** $[(M + Na) – C20:2]^+$; **544** $[(M + Na) – C20:2 + H_2O]^+$; **552** $[(M + Na) – C18:1]^+$; **570** $[(M + Na) – C18:1 + H_2O]^+$; **651** $[(M + Na) – C_5H_{14}NO_4P]^+$; **775** $[(M + Na) – C_3H_9N]^+$
12	PC 18:1/20:1	$[M + Na]^+$	836	**467** $[(M + Na) – C_3H_9N – C20:1]^+$; **495** $[(M + Na) – C_3H_9N – C18:1]^+$; **526** $[(M + Na) – C20:1]^+$; **544** $[(M + Na) – C20:1 + H_2O]^+$; **572** $[(M + Na) – C18:1 + H_2O]^+$; **653** $[(M + Na) – C_5H_{14}NO_4P]^+$; **777** $[(M + Na) – C_3H_9N]^+$
	Plasmenylphosphatidylcholine (plasmenyl-PC)			
13	plasmenyl-PC 16:0/18:0	$[M + Na]^+$	768	**563** $[(M + Na) – C_5H_{14}NO_4P – Na + H]^+$; **585** $[(M + Na) – C_5H_{14}NO_4P]^+$; **709** $[(M + Na) – C_3H_9N]^+$
14	plasmenyl-PC 18:0/18:1	$[M + Na]^+$	794	**307** $[(M + Na) – C_5H_{14}NO_4P – Na – C18:0]^+$; **611** $[(M + Na) – C_5H_{14}NO_4P]^+$; **735** $[(M + Na) – C_3H_9N]^+$
15	plasmenyl-PC 18:0/18:0	$[M + Na]^+$	796	**307** $[(M + Na) – C_5H_{14}NO_4P – Na – C18:0]^+$; **591** $[(M + Na) – C_5H_{14}NO_4P – Na + H]^+$; **613** $[(M + Na) – C_5H_{14}NO_4P]^+$; **737** $[(M + Na) – C_3H_9N]^+$
16	plasmenyl-PC 16:0/22:5	$[M + Na]^+$	814	**609** $[(M + Na) – C_5H_{14}NO_4P – Na + H]^+$; **631** $[(M + Na) – C_5H_{14}NO_4P]^+$; **755** $[(M + Na) – C_3H_9N]^+$
17	plasmenyl-PC 16:0/22:4	$[M + Na]^+$	816	**279** $[(M + Na) – C_5H_{14}NO_4P – Na – C22:4]^+$; **611** $[(M + Na) – C_5H_{14}NO_4P – Na + H]^+$; **633** $[(M + Na) – C_5H_{14}NO_4P]^+$; **757** $[(M + Na) – C_3H_9N]^+$
	Plasmenylphosphatidylethanolamine plasmenyl-PE)			
18	plasmenyl-PE 16:0/22:5	$[M + Na]^+$	772	**386** $[C16:0\ ether + C_2H_8NO_3P + Na]^+$; **631** $[(M + Na) – C_2H_8NO_4P]^+$; **649** $[(M + Na) – C_2H_8NO_4P + H_2O]^+$; **729** $[(M + Na) – C_2H_5N]^+$; **754** $[(M + Na) – H_2O]^+$
	**Negative ion mode**			
	Ceramide (Cer)			
19	Cer d18:1/16:0	$[M – H]^–$	536	**237** $[M – H – 299]^–$; **254** $[M – H – 282]^–$; **255** $[M – H – 281]^–$; **280** $[M – H – 256]^–$; **296** $[M – H – 240]^–$; **488** $[M – H – CH_2O – H_2O]^–$; **504** $[M – H – CH_3OH]^–$; **506** $[M – H – CH_2O]^–$; **518** $[M – H – H_2O]^–$

	Phosphatidic acid (PA)			
20	PA 18:0/18:1	$[M - H]^-$	701	**281** $[C18:1 - H]^-$; **283** $[C18:0 - H]^-$; **280** $[C18:0]^-$; **417** $[M - C18:0 - H]^-$; **419** $[M - C18:1 - H]^-$; **435** $[M - C18:0 + H_2O]^-$; **437** $[M - C18:1 + H_2O]^-$
	Phosphatidylethanolamine (PE)			
21	PE 16:1/16:1	$[M - H]^-$	686	**253** $[C16{:}1- H]^-$; **432** [lyso-PE(16:1) − H_2O − H]⁻; **450** [lyso-PE(16:1) − H]⁻
22	PE 16:1/18:1	$[M - H]^-$	714	**253** $[C16:1 - H]^-$; **281** $[C18:1 - H]^-$; **432** [lyso-PE(16:1) − H_2O − H]⁻; **450** [lyso-PE(16:1) − H]⁻; **460** [lyso-PE(18:1) − H_2O − H]⁻; **478** [lyso-PE(18:1) − H]⁻
23	PE 16:0/18:1	$[M - H]^-$	716	**255** $[C16:0 - H]^-$; **281** $[C18:1 - H]^-$; **434** [lyso-PE(16:0) − H_2O − H]⁻; **452** [lyso-PE(16:0) − H]⁻; **460** [lyso-PE(18:1) − H_2O − H]⁻; **478** [lyso-PE(18:1) − H]⁻
24	PE 16:0/20:4	$[M - H]^-$	738	**255** $[C16:0 - H]^-$; **303** $[C20:4 - H]^-$; **434** [lyso-PE(16:0) − H_2O − H]⁻; **452** [lyso-PE(16:0) − H]⁻; **482** [lyso-PE(20:4) − H_2O − H]⁻; **500** [lyso-PE(20:4) − H]⁻
25	PE 18:1/18:1	$[M - H]^-$	742	**281** $[C18:1 - H]^-$; **460** [lyso-PE(18:1) − H_2O − H]⁻; **478** [lyso-PE(18:1) − H]⁻
26	PE 18:0/18:1	$[M - H]^-$	744	**281** $[C18:1 - H]^-$; **283** $[C18:0 - H]^-$; **460** [lyso-PE(18:1) − H_2O − H]⁻; **462** [lyso-PE(18:0) − H_2O − H]⁻; **478** [lyso-PE(18:1) − H]⁻; **480** [lyso-PE(18:0) − H]⁻
27	PE 18:1/20:4	$[M - H]^-$	764	**281** $[C18:1 - H]^-$; **303** $[C20:4 - H]^-$; **460** [lyso-PE(18:1) − H_2O − H]⁻; **478** [lyso-PE(18:1) − H]⁻; **482** [lyso-PE(20:4) − H_2O − H]⁻; **500** [lyso-PE(20:4) − H]⁻
28	PE 18:0/22:5	$[M - H]^-$	792	**283** $[C18:0 - H]^-$; **329** $[C22:5 - H]^-$; **462** [lyso-PE(18:0) − H_2O − H]⁻; **480** [lyso-PE(18:0) − H]⁻; **508** [lyso-PE(22:5) − H_2O − H]⁻; **526** [lyso-PE(22:5) − H]⁻
29	PE 18:0/22:4	$[M - H]^-$	794	**283** $[C18:0 - H]^-$; **331** $[C22:4 - H]^-$; **462** [lyso-PE(18:0) − H_2O − H]⁻; **480** [lyso-PE(18:0) − H]⁻; **510** [lyso-PE(22:4) − H_2O − H]⁻; **528** [lyso-PE(22:4) − H]⁻
	Plasmenylphosphatidylethanolamine (plasmenyl-PE)			
30	plasmenyl-PE 16:0/18:1	$[M - H]^-$	700	**281** $[C18:1 - H]^-$; **418** [lyso-PE(16:0) − H_2O − H]⁻; **436** [lyso-PE(P-16:0) − H]⁻
31	plasmenyl-PE 16:0/20:4	$[M - H]^-$	722	**303** $[C20:4 - H]^-$; **418** [lyso-PE(16:0) − H_2O − H]⁻; **436** [lyso-PE(P-16:0) − H]⁻
32	plasmenyl-PE 18:0/18:1	$[M - H]^-$	728	**281** $[C18:1 - H]^-$; **446** [lyso-PE(18:0) − H_2O − H]⁻; **464** [lyso-PE(18:1) − H]⁻

33	plasmenyl-PE 18:0/22:5	$[M-H]^-$	776	**329** [C22:5 – H]⁻; **446** [lyso-PE(18:0) – H_2O – H]⁻; **464** [lyso-PE(18:0) – H]⁻
	Phosphatidylglycerol (PG)			
34	PG 16:1/18:1	$[M-H]^-$	745	**253** [C16:1 – H]⁻; **281** [C18:1 – H]⁻; **389** [lyso-PG(16:1) – $C_3H_6O_2$ – H]⁻; **417** [lyso-PG(18:1) – $C_3H_6O_2$ – H]⁻; **463** [lyso-PG(16:1) – H_2O – H]⁻; **481** [lyso-PG(16:1) – H]⁻; **491** [lyso-PG(18:1) – H_2O – H]⁻; **509** [lyso-PG(18:1) – H]⁻
35	PG 16:0/18:1	$[M-H]^-$	747	**255** [C16:0 – H]⁻; **281** [C18:1 – H]⁻; **391** [lyso-PG(16:0) – $C_3H_6O_2$ – H]⁻; **417** [lyso-PG(18:1) – $C_3H_6O_2$ – H]⁻; **465** [lyso-PG(16:0) – H_2O – H]⁻; **483** [lyso-PG(16:0) – H]⁻; **491** [lyso-PG(18:1) – H_2O – H]⁻; **509** [lyso-PG(18:1) – H]⁻
36	PG 18:1/18:1	$[M-H]^-$	773	**281** [C18:1 – H]⁻; **417** [lyso-PG(18:1) – $C_3H_6O_2$ – H]⁻; **491** [lyso-PG(18:1) – H_2O – H]⁻; **509** [lyso-PG(18:1) – H]⁻
37	PG 18:0/18:1	$[M-H]^-$	775	**281** [C18:1 – H]⁻; **283** [C18:0 – H]⁻; **417** [lyso-PG(18:1) – $C_3H_6O_2$ – H]⁻; **419** [lyso-PG(18:0) – $C_3H_6O_2$ – H]⁻; **491** [lyso-PG(18:1) – H_2O – H]⁻; **493** [lyso-PG(18:0) – H_2O – H]⁻; **509** [lyso-PG(18:1) – H]⁻; **511** [lyso-PG(18:0) – H]⁻
	Phosphatidylserine (PS)			
38	PS 16:0/16:1	$[M-H]^-$	732	**253** [C16:1 – H]⁻; **255** [C16:0 – H]⁻; **389** [lyso-PS(16:1) – $C_3H_5NO_2$ – H]⁻; **391** [lyso-PS(16:0) – $C_3H_5NO_2$ – H]⁻ ; **407** [lyso-PS(16:1) – $C_3H_5NO_2$ + H_2O – H]⁻; **409** [lyso-PS(16:0) – $C_3H_5NO_2$ + H_2O – H]⁻; **645** [M – $C_3H_5NO_2$ – H]⁻
39	PS 16:0/16:0	$[M-H]^-$	734	**255** [C16:0 – H]⁻; **409** [lyso-PS(16:0) – $C_3H_5NO_2$ + H_2O – H]⁻; **647** [M – $C_3H_5NO_2$ – H]⁻
40	PS 16:1/18:1	$[M-H]^-$	758	**253** [C16:1 – H]⁻; **281** [C18:1 – H]⁻; **389** [lyso-PS(16:1) – $C_3H_5NO_2$ – H]⁻; **407** [lyso-PS(16:1) – $C_3H_5NO_2$ + H_2O – H]⁻; **417** [lyso-PS(18:1) – $C_3H_5NO_2$ – H]⁻; **435** [lyso-PS(18:1) – $C_3H_5NO_2$ + H_2O – H]⁻; **671** [M – $C_3H_5NO_2$ – H]⁻
41	PS 16:0/18:1	$[M-H]^-$	760	**225** [C16:0 – H]⁻; **281** [C18:1 – H]⁻; **391** [lyso-PS(16:0) – $C_3H_5NO_2$ – H]⁻; **409** [lyso-PS(16:0) – $C_3H_5NO_2$ + H_2O – H]⁻; **417** [lyso-PS(18:1) – $C_3H_5NO_2$ – H]⁻; **435** [lyso-PS(18:1) – $C_3H_5NO_2$ + H_2O – H]⁻; **673** [M – $C_3H_5NO_2$ – H]⁻
42	PS 16:0/18:0	$[M-H]^-$	762	**255** [C16:0 – H]⁻; **283** [C18:0 – H]⁻; **391** lyso-PS(16:0) – $C_3H_5NO_2$ – H]⁻ ; **409** [lyso-PS(16:0) – $C_3H_5NO_2$ + H_2O – H]⁻; **419** [lyso-PS(18:0) – $C_3H_5NO_2$ – H]⁻; **437** [lyso-PS(18:0) – $C_3H_5NO_2$ + H_2O – H]⁻; **675** [M – $C_3H_5NO_2$ – H]⁻

43	PS 18:1/18:2	$[M-H]^-$	784	**279** [C18:2 – H]⁻; **281** [C18:1 – H]⁻; **415** [lyso-PS(18:2) – $C_3H_5NO_2$ – H]⁻ ; **417** [lyso-PS(18:1) $C_3H_5NO_2$ – H]⁻; **433** [lyso-PS(18:2) – $C_3H_5NO_2$ + H_2O – H]⁻; **435** [lyso-PS(18:1) – $C_3H_5NO_2$ + H_2O – H]⁻; **697** [M – $C_3H_5NO_2$ – H]⁻
44	PS 18:1/18:1	$[M-H]^-$	786	**281** [C18:1 – H]⁻; **417** [lyso-PS(18:1) – $C_3H_5NO_2$ – H]⁻; **435** [lyso-PS(18:1) – $C_3H_5NO_2$ + H_2O – H]⁻; **699** [M – $C_3H_5NO_2$ – H]⁻
45	PS 18:0/18:1	$[M-H]^-$	788	**281** [C18:1 – H]⁻; **283** [C18:0 – H]⁻; **417** [lyso-PS(18:1) $C_3H_5NO_2$ – H]⁻; **419** [lyso-PS(18:0) – $C_3H_5NO_2$ – H]⁻; **435** [lyso-PS(18:1) – $C_3H_5NO_2$ + H_2O – H]⁻; **437** [lyso-PS(18:0)) – $C_3H_5NO_2$ + H_2O – H]⁻; **701** [M – $C_3H_5NO_2$ – H]⁻
46	PS 18:0/18:0	$[M-H]^-$	790	**283** [C18:0 – H]⁻; **419** [lyso-PS(18:0) – $C_3H_5NO_2$ – H]⁻; **437** [lyso-PS(18:0) – $C_3H_5NO_2$ + H_2O – H]⁻; **703** [M – $C_3H_5NO_2$ – H]⁻
47	PS 18:1/20:2	$[M-H]^-$	812	**283** [C18:0 – H]⁻; **305** [C20:3 – H]⁻; **419** [lyso-PS(18:0) – $C_3H_5NO_2$ – H]⁻; **437** [lyso-PS(18:0) – $C_3H_5NO_2$ + H_2O – H]⁻; **441** [lyso-PS(20:3) – $C_3H_5NO_2$ – H]⁻; **459** [lyso-PS(20:3) – $C_3H_5NO_2$ + H_2O – H]⁻; **725** [M – $C_3H_5NO_2$ – H]⁻
48	PS 18:1/20:1	$[M-H]^-$	814	**281** [C18:1 – H]⁻; **309** [C20:1 – H]⁻; **417** [lyso-PS(18:1) – $C_3H_5NO_2$ – H]⁻; **435** [lyso-PS(18:1) – $C_3H_5NO_2$ + H_2O – H]⁻; **445** [lyso-PS(20:1) – $C_3H_5NO_2$ – H]⁻; **463** [lyso-PS(20:1) – $C_3H_5NO_2$ + H_2O – H]⁻; **727** [M – $C_3H_5NO_2$ – H]⁻
49	PS 18:1/20:0	$[M-H]^-$	816	**281** [C18:1 – H]⁻; **311** [C20:0 – H]⁻; **417** [lyso-PS(18:1) – $C_3H_5NO_2$ – H]⁻; **435** [lyso-PS(18:1) – $C_3H_5NO_2$ + H_2O – H]⁻; **447** [lyso-PS(20:0) – $C_3H_5NO_2$ – H]⁻; **465** [lyso-PS(20:0) – $C_3H_5NO_2$ + H_2O – H]⁻; **729** [M – $C_3H_5NO_2$ – H]⁻
50	PS 18:1/22:6	$[M-H]^-$	832	**281** [C18:1 – H]⁻; **337** [C22:6 – H]⁻; **417** [lyso-PS(18:1) – $C_3H_5NO_2$ – H]⁻; **435** [lyso-PS(18:1) – $C_3H_5NO_2$ + H_2O – H]⁻; **463** [lyso-PS(22:6) – $C_3H_5NO_2$ – H]⁻; **481** [lyso-PS(22:6) – $C_3H_5NO_2$ + H_2O – H]⁻; **745** [M – $C_3H_5NO_2$ – H]⁻
51	PS 18:1/22:1	$[M-H]^-$	842	**281** [C18:1 – H]⁻; **337** [C22:1 – H]⁻; **417** [lyso-PS(18:1) – $C_3H_5NO_2$ – H]⁻; **435** [lyso-PS(18:1) – $C_3H_5NO_2$ + H_2O – H]⁻; **473** [lyso-PS(22:1) – $C_3H_5NO_2$ – H]⁻; **491** [lyso-PS(22:1) – $C_3H_5NO_2$ + H_2O – H]⁻; **755** [M – $C_3H_5NO_2$ – H]⁻
52	PS 18:1/22:0	$[M-H]^-$	844	**281** [C18:1 – H]⁻; **339** [C22:0 – H]⁻; **417** [lyso-PS(18:1) – $C_3H_5NO_2$ – H]⁻; **435** [lyso-PS(18:1) – $C_3H_5NO_2$ + H_2O – H]⁻; **475** [lyso-PS(22:0) – $C_3H_5NO_2$ – H]⁻; **493** [lyso-PS(22:0) – $C_3H_5NO_2$ + H_2O – H]⁻; **757** [M – $C_3H_5NO_2$ – H]⁻

Phosphatidylinositol (PI)

53	PI 16:0/16:1	$[M-H]^-$	807	**253** [C16:1 – H]⁻; **255** [C16:0 – H]⁻; **389** [lyso-PI(16:1) – $C_6H_{12}O_6$ – H]⁻; **391** [lyso-PI(16:0) – $C_6H_{12}O_6$ – H]⁻; **551** [lyso-PI(16:1) – H_2O – H]⁻; **553** [lyso-PI(16:0) – H_2O – H]⁻; **569** [lyso-PI(16:1) – H]⁻; **571** [lyso-PI(16:0) – H]⁻
54	PI 16:1/18:1	$[M-H]^-$	833	**253** [C16:1 – H]⁻; **281** [C18:1 – H]⁻; **389** [lyso-PI(16:1) – $C_6H_{12}O_6$ – H]⁻; **417** [lyso-PI(18:1) – $C_6H_{12}O_6$ – H]⁻; **551** [lyso-PI(16:1) – H_2O – H]⁻; **569** [lyso-PI(16:1) – H]⁻; **579** [lyso-PI(18:1) – H_2O – H]⁻; **597** [lyso-PI(18:1) – H]⁻
55	PI 16:0/18:1	$[M-H]^-$	835	**255** [C16:0 – H]⁻; **281** [C18:1 – H]⁻; **391** [lyso-PI(16:0) – $C_6H_{12}O_6$ – H]⁻; **417** [lyso-PI(18:1) – $C_6H_{12}O_6$ – H]⁻; **553** [lyso-PI(16:0) – H_2O – H]⁻; **571** [lyso-PI(16:0) – H]⁻; **579** [lyso-PI(18:1) – H_2O – H]⁻ ; **597** [lyso-PI(18:1) – H]⁻
56	PI 16:0/20:3	$[M-H]^-$	859	**279** [C18:2 – H]⁻; **281** [C18:1 – H]⁻; **415** [lyso-PI(18:2) – $C_6H_{12}O_6$ – H]⁻; **417** [lyso-PI(18:1) – $C_6H_{12}O_6$ – H]⁻; **577** [lyso-PI(18:2) – H_2O – H]⁻; **579** [lyso-PI(18:1) – H_2O – H]⁻; **595** [lyso-PI(18:2) – H]⁻; **597** [lyso-PI(18:1) – H]⁻
57	PI 18:1/18:1	$[M-H]^-$	861	**281** [C18:1 – H]⁻; **417** [lyso-PI(18:1) – $C_6H_{12}O_6$ – H]⁻; **579** [lyso-PI(18:1) – H_2O – H]⁻; **597** [lyso-PI(18:1) – H]⁻
58	PI 18:0/18:1	$[M-H]^-$	863	**281** [C18:1 – H]⁻; **283** [C18:0 – H]⁻; **417** [lyso-PI(18:1) – $C_6H_{12}O_6$ – H]⁻; **419** [lyso-PI(18:0) – $C_6H_{12}O_6$ – H]⁻; **579** [lyso-PI(18:1) – H_2O – H]⁻; **581** [lyso-PI(18:0) – H_2O – H]⁻; **597** [lyso-PI(18:1) – H]⁻ ; **599** [lyso-PI(18:0) – H]⁻
59	PI 18:1/20:4	$[M-H]^-$	883	**281** [C18:1 – H]⁻; **303** [C20:4 – H]⁻; **417** [lyso-PI(18:1) – $C_6H_{12}O_6$ – H]⁻; **439** [lyso-PI(20:4) – $C_6H_{12}O_6$ – H]⁻; **579** [lyso-PI(18:1) – H_2O – H]⁻; **597** [lyso-PI(18:1) – H]⁻ ; **601** [lyso-PI(20:4) – H_2O – H]⁻; **619** [lyso-PI(20:4) – H]⁻
60	PI 18:0/20:4	$[M-H]^-$	885	**283** [C18:0 – H]⁻; **303** [C20:4 – H]⁻; **419** [lyso-PI(18:0) – $C_6H_{12}O_6$ – H]⁻; **439** [lyso-PI(20:4) – $C_6H_{12}O_6$ – H]⁻; **581** [lyso-PI(18:0) – H_2O – H]⁻; **599** [lyso-PI(18:0) – H]⁻; **601** [lyso-PI(20:4) – H_2O – H]⁻; **619** [lyso-PI(20:4) – H]⁻
61	PI 18:0/20:3	$[M-H]^-$	887	**283** [C18:0 – H]⁻; **305** [C20:3 – H]⁻; **419** [lyso-PI(18:0) – $C_6H_{12}O_6$ – H]⁻; **441** [lyso-PI(20:3) – $C_6H_{12}O_6$ – H]⁻; **581** [lyso-PI(18:0) – H_2O – H]⁻; **599** [lyso-PI(18:0) – H]⁻; **603** [lyso-PI(20:3) – H_2O – H]⁻; **621** [lyso-PI(20:3) – H]⁻
62	PI 18:0/20:2	$[M-H]^-$	889	**283** [C18:0 – H]⁻; **307** [C20:2 – H]⁻; **419** [lyso-PI(18:0) – $C_6H_{12}O_6$ – H]⁻; **443** [lyso-PI(20:2) – $C_6H_{12}O_6$ – H]⁻; **581** [lyso-PI(18:0) – H_2O – H]⁻; **599** [lyso-PI(18:0) – H]⁻; **605** [lyso-PI(20:2) – H_2O – H]⁻; **623** [lyso-PI(20:2) – H]⁻

Table S4. Relative levels of identified lipids in HBEpC and lung SQCC cell lines (H520, HCC95, SK-MES-1, and H1703) analyzed by DI-MS.

No.	Lipid species	Ion species	m/z	Normal	Low metastatic potential		High metastatic potential	
				HBEpC	H520	HCC95	SK-MES-1	H1703
	**Positive ion mode**							
	Phosphatidylcholine (PC)							
1	PC 16:0/16:1	$[M + H]^+$	732	107.82 ± 26.72[a]	82.99 ± 3.58 [ab]	156.71 ± 28.08[c]	73.53 ± 7.23[b]	107.62 ± 30.86[a]
2	PC 16:0/16:0	$[M + H]^+$	734	43.14 ± 9.10[a]	28.93 ± 2.01[b]	93.90 ± 14.99[c]	50.00 ± 4.03[a]	46.08 ± 11.68[a]
3	PC 16:1/18:1	$[M + H]^+$	758	92.15 ± 22.32[a]	46.52 ± 2.78[b]	144.28 ± 25.83[b]	55.43 ± 5.14[c]	113.25 ± 34.36[a]
4	PC 16:0/18:1	$[M + H]^+$	760	179.71 ± 41.63[a]	207.93 ± 9.61[ab]	337.41 ± 53.89[c]	250.88 ± 26.42[bd]	285.01 ± 74.74[cd]
5	PC 16:0/18:0	$[M + H]^+$	762	36.58 ± 9.33[a]	36.21 ± 3.40[a]	55.69 ± 5.26[b]	45.27 ± 4.60[ab]	38.93 ± 12.04[a]
6	PC 18:1/18:1	$[M + H]^+$	786	252.37 ± 56.34[a]	204.70 ± 46.11[a]	344.86 ± 24.16[b]	198.44 ± 25.34[a]	264.90 ± 90.18[a]
7	PC 18:0/18:1	$[M + H]^+$	788	95.79 ± 22.45[ac]	71.44 ± 12.15[a]	152.38 ± 13.32[b]	126.40 ± 16.99[bc]	92.00 ± 39.54[c]
8	PC 16:1/20:3	$[M + Na]^+$	804	61.22 ± 23.78[a]	54.35 ± 13.28[abc]	58.22 ± 13.50[ab]	36.33 ± 2.16[bc]	33.32 ± 18.45[c]
9	PC 18:0/18:0	$[M + Na]^+$	812	8.84 ± 2.15[a]	5.81 ± 0.20[b]	14.97 ± 3.51[c]	8.25 ± 0.83[ab]	6.14 ± 1.98[ab]
10	PC 18:0/20:4	$[M + Na]^+$	832	10.15 ± 7.69[a]	7.97 ± 0.61[a]	17.20 ± 2.31[b]	12.77 ± 1.00[ab]	13.92 ± 5.94[ab]
11	PC 18:1/20:2	$[M + Na]^+$	834	7.54 ± 4.41[ac]	6.65 ± 0.58[a]	13.43 ± 2.26[b]	11.01 ± 1.25[bc]	10.52 ± 3.86[abc]

12	PC 18:1/20:1	[M + Na]+	836	1.58 ± 0.42[a]	3.67 ± 0.26[b]	8.66 ± 1.71[c]	7.28 ± 1.01[c]	8.91 ± 2.29[c]
	Plasmenylphosphatidylcholine (plasmenyl-PC)							
13	plasmenyl-PC 16:0/18:0	[M + Na]+	768	8.45 ± 1.99[a]	19.10 ± 1.26[b]	17.00 ± 2.56[b]	23.36 ± 2.39[c]	12.97 ± 2.27[d]
14	plasmenyl-PC 18:0/18:1	[M + Na]+	794	2.37 ± 0.73[a]	8.61 ± 0.49[b]	4.24 ± 1.31[c]	11.31 ± 1.27[b]	8.36 ± 1.49[d]
15	plasmenyl-PC 18:0/18:0	[M + Na]+	796	12.69 ± 5.04[ab]	10.81 ± 0.98[ab]	8.83 ± 1.44[a]	14.47 ± 1.77[b]	10.40 ± 4.88[ab]
16	plasmenyl-PC 16:0/22:5	[M + Na]+	814	16.20 ± 5.07[a]	15.75 ± 2.00[a]	42.58 ± 6.46[b]	14.98 ± 1.18[a]	25.99 ± 6.41[c]
17	plasmenyl-PC 16:0/22:4	[M + Na]+	816	11.61 ± 5.36[ab]	11.36 ± 2.30[ab]	15.52 ± 0.99[b]	8.28 ± 0.33[a]	8.11 ± 3.55[a]
	Plasmenylphosphatidylethanolamine (plasmenyl-PE)							
18	plasmenyl-PE 16:0/22:5	[M + Na]+	772	11.60 ± 2.83[a]	20.07 ± 1.03[bd]	18.36 ± 1.90[b]	32.98 ± 2.44[c]	23.60 ± 6.28[d]
	**Negative ion mode**							
	Ceramide (Cer)							
19	Cer d18:1/16:0	[M − H]−	536	270.28 ± 50.97[a]	51.45 ± 6.77[b]	84.66 ± 26.10[bc]	96.13 ± 14.47[c]	79.24 ± 31.28[bc]
	Phosphatidic acid (PA)							
20	PA 18:0/18:1	[M − H]−	701	16.95 ± 2.44[a]	10.46 ± 0.74[b]	3.32 ± 1.30[c]	3.32 ± 1.30[d]	4.00 ± 0.44[c]
	Phosphatidylethanolamine (PE)							

21	PE 16:1/16:1	[M − H]⁻	686	2.30 ± 0.48[ab]	1.55 ± 0.34[ac]	2.86 ± 0.63[b]	1.34 ± 0.24[c]	2.52 ± 0.78[b]
22	PE 16:1/18:1	[M − H]⁻	714	12.91 ± 1.88[a]	7.86 ± 0.80[b]	22.73 ± 3.60[c]	8.38 ± 0.94[b]	12.03 ± 1.25[a]
23	PE 16:0/18:1	[M − H]⁻	716	17.88 ± 2.45[ac]	10.19 ± 0.76[b]	20.45 ± 3.05[c]	15.17 ± 1.42[a]	16.87 ± 1.30[a]
24	PE 16:0/20:4	[M − H]⁻	738	1.74 ± 0.52[a]	2.20 ± 0.24[ab]	5.74 ± 1.53[c]	2.84 ± 0.35[b]	1.85 ± 0.34[ab]
25	PE 18:1/18:1	[M − H]⁻	742	40.71 ± 6.28[a]	17.66 ± 1.64[b]	51.11 ± 7.28[c]	32.11 ± 2.44[d]	43.73 ± 3.61[a]
26	PE 18:0/18:1	[M − H]⁻	744	26.80 ± 4.36[a]	27.58 ± 2.31[a]	19.94 ± 2.63[b]	45.12 ± 4.45[c]	18.09 ± 2.85[b]
27	PE 18:1/20:4	[M − H]⁻	764	16.85 ± 2.91[a]	14.23 ± 1.24[a]	26.18 ± 3.17[b]	22.35 ± 2.01[c]	16.04 ± 1.08[a]
28	PE 18:0/22:5	[M − H]⁻	792	17.02 ± 2.84[a]	25.45 ± 2.90[b]	41.83 ± 4.81[c]	33.91 ± 3.15[d]	25.33 ± 1.99[b]
29	PE 18:0/22:4	[M − H]⁻	794	5.29 ± 1.55[a]	6.87 ± 0.82[a]	12.54 ± 4.08[b]	11.32 ± 1.93[b]	7.95 ± 0.66[a]
	Plasmenylphosphatidylethanolamine (plasmenyl-PE)							
30	plasmenyl-PE 16:0/18:1	[M − H]⁻	700	44.00 ± 6.38[a]	19.68 ± 1.61[b]	3.65 ± 1.20[c]	15.71 ± 0.97[b]	10.35 ± 1.05[d]
31	plasmenyl-PE 16:0/20:4	[M − H]⁻	722	9.69 ± 1.87[a]	9.50 ± 0.51[a]	5.14 ± 0.97[b]	19.30 ± 1.34[c]	8.79 ± 1.09[a]
32	plasmenyl-PE 18:0/18:1	[M − H]⁻	728	9.23 ± 1.34[a]	8.96 ± 0.49[a]	1.88 ± 0.62[b]	12.47 ± 1.06[c]	3.87 ± 0.52[d]
33	plasmenyl-PE 18:0/22:5	[M − H]⁻	776	22.16 ± 5.14[a]	6.73 ± 0.25[b]	32.32 ± 7.12[c]	51.23 ± 3.09[d]	17.97 ± 5.10[a]
	Phosphatidylglycerol (PG)							
34	PG 16:1/18:1	[M − H]⁻	745	26.27 ± 3.57[a]	16.88 ± 2.34[b]	26.26 ± 7.70[a]	34.56 ± 1.76[c]	18.19 ± 5.74[b]

35	PG 16:0/18:1	[M − H]⁻	747	30.43 ± 4.25^{a}	39.29 ± 4.43^{ab}	49.30 ± 15.49^{b}	52.87 ± 3.45^{b}	50.84 ± 14.61^{b}
36	PG 18:1/18:1	[M − H]⁻	773	61.60 ± 8.52^{a}	16.41 ± 4.03^{b}	25.68 ± 8.36^{bd}	98.24 ± 6.97^{c}	35.58 ± 16.27^{d}
37	PG 18:0/18:1	[M − H]⁻	775	20.75 ± 2.98^{a}	38.41 ± 4.30^{b}	17.77 ± 4.10^{a}	60.17 ± 3.56^{c}	17.52 ± 7.90^{a}
	Phosphatidylserine (PS)							
38	PS 16:0/16:1	[M − H]⁻	732	33.11 ± 7.25^{a}	11.35 ± 0.83^{b}	18.37 ± 3.30^{c}	12.44 ± 2.31^{b}	8.46 ± 1.69^{b}
39	PS 16:0/16:0	[M − H]⁻	734	16.06 ± 3.25^{a}	9.41 ± 0.75^{bc}	15.19 ± 2.72^{a}	9.73 ± 1.81^{b}	6.50 ± 1.35^{c}
40	PS 16:1/18:1	[M − H]⁻	758	73.69 ± 15.09^{a}	31.85 ± 2.42^{b}	54.99 ± 8.89^{c}	35.90 ± 5.83^{b}	33.01 ± 7.33^{b}
41	PS 16:0/18:1	[M − H]⁻	760	131.37 ± 26.20^{a}	102.63 ± 6.28^{b}	115.20 ± 21.63^{ab}	120.63 ± 17.98^{ab}	66.34 ± 15.80^{c}
42	PS 16:0/18:0	[M − H]⁻	762	35.57 ± 6.51^{a}	26.24 ± 1.76^{bc}	35.33 ± 3.17^{a}	31.15 ± 4.71^{ab}	21.31 ± 3.64^{c}
43	PS 18:1/18:2	[M − H]⁻	784	39.64 ± 8.18^{a}	17.61 ± 1.31^{bd}	25.59 ± 4.44^{c}	24.54 ± 4.34^{bc}	16.45 ± 4.24^{d}
44	PS 18:1/18:1	[M − H]⁻	786	190.44 ± 37.22^{a}	103.28 ± 5.79^{b}	174.14 ± 30.49^{a}	184.41 ± 28.33^{a}	122.80 ± 30.84^{b}
45	PS 18:0/18:1	[M − H]⁻	788	126.52 ± 21.62^{a}	135.92 ± 6.92^{a}	117.82 ± 21.97^{ab}	303.47 ± 24.24^{c}	89.88 ± 27.25^{b}
46	PS 18:0/18:0	[M − H]⁻	790	41.87 ± 6.56^{a}	56.72 ± 3.35^{b}	65.67 ± 6.94^{c}	71.62 ± 7.45^{c}	44.80 ± 4.69^{a}
47	PS 18:1/20:2	[M − H]⁻	812	11.90 ± 2.07^{ac}	6.69 ± 0.50^{b}	10.67 ± 2.14^{a}	13.73 ± 1.14^{c}	5.81 ± 1.58^{b}
48	PS 18:1/20:1	[M − H]⁻	814	14.28 ± 2.32^{a}	13.57 ± 0.65^{a}	27.39 ± 4.66^{b}	17.48 ± 1.36^{a}	25.66 ± 4.95^{b}

49	PS 18:1/20:0	$[M-H]^-$	816	23.32 ± 4.02^{a}	22.35 ± 1.36^{a}	43.29 ± 5.54^{b}	30.43 ± 2.78^{c}	35.44 ± 3.30^{c}
50	PS 18:1/22:6	$[M-H]^-$	832	6.50 ± 1.92^{a}	13.68 ± 0.98^{b}	16.92 ± 3.14^{b}	29.42 ± 3.05^{c}	13.78 ± 1.72^{b}
51	PS 18:1/22:1	$[M-H]^-$	842	9.72 ± 1.65^{a}	14.11 ± 0.50^{b}	19.65 ± 2.83^{c}	13.05 ± 1.03^{b}	17.63 ± 2.79^{c}
52	PS 18:1/22:0	$[M-H]^-$	844	25.93 ± 4.55^{a}	35.90 ± 2.17^{b}	67.65 ± 9.15^{c}	63.58 ± 6.98^{cd}	56.31 ± 5.49^{d}
	Phosphatidylinositol (PI)							
53	PI 16:0/16:1	$[M-H]^-$	807	3.50 ± 1.14^{a}	5.82 ± 1.58^{ab}	11.92 ± 3.44^{c}	11.79 ± 2.16^{c}	7.83 ± 1.60^{b}
54	PI 16:1/18:1	$[M-H]^-$	833	15.17 ± 2.80^{a}	17.16 ± 0.91^{a}	32.54 ± 11.16^{b}	31.34 ± 2.29^{b}	37.49 ± 8.15^{b}
55	PI 16:0/18:1	$[M-H]^-$	835	16.77 ± 2.98^{a}	25.15 ± 1.80^{ab}	59.79 ± 25.57^{c}	46.50 ± 3.63^{cd}	38.97 ± 8.43^{bd}
56	PI 16:0/20:3	$[M-H]^-$	859	27.64 ± 5.19^{a}	35.84 ± 1.95^{b}	17.85 ± 4.99^{c}	27.79 ± 1.37^{a}	16.42 ± 3.47^{c}
57	PI 18:1/18:1	$[M-H]^-$	861	50.69 ± 9.36^{a}	51.71 ± 4.23^{a}	67.86 ± 26.51^{a}	100.72 ± 6.93^{b}	105.32 ± 22.28^{b}
58	PI 18:0/18:1	$[M-H]^-$	863	24.42 ± 4.45^{a}	26.32 ± 1.91^{a}	61.60 ± 27.50^{bc}	77.73 ± 8.74^{c}	56.09 ± 13.34^{b}
59	PI 18:1/20:4	$[M-H]^-$	883	8.06 ± 1.41^{a}	28.46 ± 2.60^{b}	21.55 ± 8.45^{b}	47.54 ± 3.09^{c}	49.06 ± 11.40^{c}
60	PI 18:0/20:4	$[M-H]^-$	885	56.15 ± 10.03^{a}	162.61 ± 12.32^{b}	51.97 ± 19.66^{a}	175.88 ± 9.91^{b}	70.54 ± 20.65^{a}
61	PI 18:0/20:3	$[M-H]^-$	887	128.24 ± 25.24^{a}	159.75 ± 12.57^{b}	51.92 ± 20.37^{c}	175.95 ± 7.86^{b}	36.60 ± 17.48^{c}
62	PI 18:0/20:2	$[M-H]^-$	889	70.79 ± 13.49^{a}	56.91 ± 4.32^{ab}	43.60 ± 18.47^{bc}	63.05 ± 3.46^{a}	30.41 ± 8.53^{c}

Each value represents the mean ± SD (n = 8). Significant differences were evaluated using ANOVA with a Tukey's *post-hoc* test and different letters represent statistically significant differences among samples ($p < 0.05$).

Table S5. Metabolites and intact lipid species with variable influence on projection (VIP) values higher than 1.0.

No.	Normal vs. Low metastatic potential		Normal vs. High metastatic potential		Low vs. High metastatic potential	
	Compound	VIP[3]	Compound	VIP[2]	Compound	VIP[2]
1	Lactose	1.570	Aspartic acid	1.441	Linoleic acid	2.103
2	Cer d18:1/16:0	1.568	plasmenyl-PE 16:0/18:1	1.433	Aspartic acid	2.064
3	PG 18:1/18:1	1.546	Lactic acid	1.419	*myo*-inositol	2.044
4	Ribitol	1.536	PA 18:0/18:1	1.414	Aminomalonic acid	1.945
5	plasmenyl-PC 16:0/18:0	1.515	1-Monopalmitin	1.407	PI 18:1/20:4	1.917
6	plasmenyl-PE 16:0/18:1	1.497	PI 18:1/20:4	1.407	Hypoxanthine	1.883
7	Glyceric acid	1.485	Hypoxanthine	1.406	Inosine	1.814
8	Inosine	1.484	PS 18:1/22:0	1.388	PI 18:1/18:1	1.760
9	Linoleic acid	1.481	Cer d18:1/16:0	1.385	β-Alanine	1.664
10	Glycerol	1.470	Valine	1.378	plasmenyl-PC 16:0/22:4	1.636
11	plasmenyl-PE 16:0/22:5	1.452	PS 16:0/16:1	1.372	Glycerol-3-phosphate	1.622
12	Succinic acid	1.442	Nicotinamide	1.359	Uracil	1.602
13	Aminomalonic acid	1.435	*myo*-inositol	1.357	plasmenyl-PE 16:0/22:5	1.575
14	PS 18:1/22:6	1.434	Ribitol	1.351	PG 18:1/18:1	1.561
15	Nicotinamide	1.431	plasmenyl-PC 18:0/18:1	1.347	Uridine	1.504
16	PS 16:0/16:1	1.428	β-Alanine	1.342	Alanine	1.498
17	Arachidonic acid	1.423	Tyrosine	1.342	PC 16:1/20:3	1.479
18	1-Monopalmitin	1.418	Aminomalonic acid	1.341	Malic acid	1.476
19	Hydroxyproline	1.403	11-Eicosenoic acid	1.339	plasmenyl-PE 16:0/20:4	1.456
20	Putrescine	1.402	Tryptophan	1.334	plasmenyl-PC 18:0/18:1	1.421
21	Phenylalanine	1.399	PI 16:0/18:1	1.334	Lactic acid	1.367

22	Creatinine	1.378	PC 18:1/20:1	1.331	Glycine	1.354
23	Erythrose	1.375	Glycerol	1.330	Fumaric acid	1.339
24	PA 18:0/18:1	1.367	PS 16:1/18:1	1.320	PS 16:0/16:0	1.331
25	PI 18:1/20:4	1.366	Ribose	1.319	1-Monopalmitin	1.249
26	PS 18:1/18:2	1.361	Erythrose	1.302	Glucose	1.237
27	PS 18:0/18:0	1.352	PI 18:1/18:1	1.299	PS 16:0/16:1	1.201
28	PS 18:1/22:1	1.270	Inosine	1.291	Cysteine	1.201
29	*myo*-inositol	1.245	PI 16:1/18:1	1.275	Proline	1.177
30	11-Eicosenoic acid	1.243	Lactose	1.267	PI 16:1/18:1	1.113
31	Pyroglutamic acid	1.233	PI 18:0/18:1	1.263	Palmitic acid	1.109
32	PS 16:1/18:1	1.220	Linoleic acid	1.258	PI 18:0/18:1	1.092
33	Cysteine	1.213	Phenylalanine	1.255	Lactose	1.086
34	Aspartic acid	1.212	Cysteine	1.244	PE 16:0/20:4	1.078
35	PE 18:0/22:5	1.209	Putrescine	1.244	PS 18:1/22:6	1.061
36	Valine	1.192	PS 16:0/16:0	1.240	Asparagine	1.036
37	Leucine	1.191	Methionine	1.227	Erythrose	1.035
38	Methionine	1.187	Arachidonic acid	1.223	Pyroglutamic acid	1.026
39	plasmenyl-PC 18:0/18:1	1.164	plasmenyl-PE 16:0/22:5	1.223	Lysine	1.014
40	β-Alanine	1.161	Threonine	1.222		
41	PC 18:1/20:1	1.158	PS 18:1/18:2	1.219		
42	Threonic acid	1.158	PE 18:0/22:5	1.205		
43	Uridine	1.150	PI 16:0/16:1	1.190		
44	Lysine	1.141	Succinic acid	1.151		
45	Arabitol	1.134	PS 18:1/20:0	1.143		
46	Palmitic acid	1.117	PG 16:0/18:1	1.141		

47	*myo*-inositol-1-phosphate	1.114	Palmitic acid	1.134
48	Oleic acid	1.113	Pyroglutamic acid	1.130
49	Tyrosine	1.102	Leucine	1.108
50	PS 18:1/22:0	1.096	Uridine	1.105
51	Proline	1.050	PS 18:1/22:6	1.087
52	PI 16:0/16:1	1.030	PE 18:0/22:4	1.084
53			PS 18:1/22:1	1.059
54			Oleic acid	1.058
55			Isoleucine	1.045
56			plasmenyl-PC 16:0/18:0	1.045
57			Malic acid	1.042
58			Fumaric acid	1.005

VIP[2], VIP values obtained from PLS models built by 2 components of PLS; VIP[3], VIP values obtained from PLS models built by 3 components of PLS.
